# Impact of wheat GRF4-GIF1 morphogenic regulators on transformation and genome editing efficiency in elite barley cultivars

**DOI:** 10.3389/fpls.2026.1837794

**Published:** 2026-08-31

**Authors:** Ekaterina M. Timonova, Antonina A. Kiseleva, Mihail A. Nesterov, Anna V. Korotkova, Irina G. Adonina, Alex V. Kochetov, Elena A. Salina

**Affiliations:** 1 The Federal State Budgetary Institution of Science Federal Research Center Institute of Cytology and Genetics, Siberian Branch of the Russian Academy of Sciences, Novosibirsk, Russia; 2 Kurchatov Genomics Center, Institute of Cytology and Genetics SB RAS, Novosibirsk, Russia

**Keywords:** biolistics, CRISPR/Cas9, genome editing, genotype independence, GRF4-GIF1, morphogenic regulators, TransIT-2020

## Abstract

**Introduction:**

Efficient genetic transformation is essential for the delivery of the CRISPR/Cas9 genome editing system and thus represents an important technology for breeding-oriented research in barley (*Hordeum vulgare* L.). However, transformation and plant regeneration from tissue culture remain challenging in non-model barley genotypes. Previous studies demonstrated that expression of a chimeric fusion between two interacting transcription factors, GROWTH-REGULATING FACTOR 4 (GRF4) and GRF-INTERACTING FACTOR 1 (GIF1), enhances regeneration capacity in wheat and other species.

**Methods:**

In this study, we evaluated the effect of the wheat-derived *GRF4-GIF1* morphogenic regulators on biolistic transformation and genome editing efficiency in three commercial barley cultivars: Tselinniy 5, Aley, and G-23035.

**Results:**

The JD633 construct carrying GRF4-GIF1 enabled recovery of stable transformants in all three genotypes, with efficiencies ranging from 2.5% to 5%, whereas the control construct lacking morphogenic regulators resulted in no transgenic events in any of the tested varieties. Among transformed T^0^ plantlets, genome editing efficiency reached 64.3%, with predominantly biallelic mutations that were stably inherited in the T^1^ generation. Molecular screening revealed the presence of plasmid-free edited plants in the T^0^ generation, likely arising from transient *Cas9* expression, and provided evidence of tissue chimerism.

**Discussion:**

These results demonstrate that the *GRF-GIF* system facilitates genome editing, providing a practical framework for accelerating precision breeding in barley.

## 1 Introduction

Climate change, shifting cultivation conditions, and increased food demands require rapid development of adapted cereal crop varieties. Modern molecular and biotechnological breeding strategies provide powerful tools to accelerate this process, including marker-assisted selection, genome editing, genomic selection, and doubled haploid technologies (Naqvi et al., 2022). Among these, CRISPR/Cas-mediated genome editing enables direct modification of elite cultivars and promising breeding lines, accelerating trait improvement while increasing precision and targeting efficiency (Chen et al., 2019; Pixley et al., 2022).

Barley (*Hordeum vulgare* L.) is the fourth most widely cultivated cereal crop after wheat, rice, and maize, with annual global production exceeding 140 million tons (FAOSTAT, 2024; available at: https://www.fao.org/faostat/en/#data/QCL; accessed on 10.07.2026). Its high starch and protein content supports diverse applications in brewing and food production and animal feed (Harwood, 2019). Because barley has a diploid genome (2n = 14), is predominantly self-pollinating, and is well characterized genetically, it provides a tractable system for studying gene function and regulatory networks. These features, together with the availability of extensive genetic and genomic resources, make barley a particularly suitable species for applying advanced genome editing technologies.

The availability of high-quality barley genome assemblies and extensive annotation of potential target genes has created a strong framework for applying genome editing technologies to accelerate development of improved varieties (Mascher et al., 2017; Milner et al., 2019; Monat et al., 2019). CRISPR/Cas9-mediated genome editing is now well established in barley, with more than 120 studies reporting targeted gene modifications (Ahmar et al., 2023). The first application targeted *HvPM19*, encoding an ABA-inducible plasma membrane protein associated with of grain dormancy in wheat (Lawrenson et al., 2015). Since then, genome editing efforts have increasingly focused on traits linked to biotic and abiotic stress resistance and yield improvement. For instance, simultaneous knockout of *HvMORC1* and *HvMORC6a* enhanced resistance to fungal pathogens such as *Blumeria graminis* and *Fusarium graminearum* (Galli et al., 2022), while mutations of *HvARE1* promoted improved nitrogen utilization, increased tiller number, and enhanced yield under nutrient-limited conditions (Karunarathne et al., 2022).

Despite substantial progress in barley genome editing, most studies have been conducted using the model cultivar Golden Promise because of its exceptional regeneration capacity and high amenability to Agrobacterium-mediated transformation (Hensel et al., 2008; Lim et al., 2018; Han et al., 2021; Hanak et al., 2023; Orman-Ligeza et al., 2020). In contrast, translation of CRISPR/Cas technologies to commercial cultivars remains limited by the strong genotype dependence of regeneration and Agrobacterium-mediated transformation, for which efficient protocols are still lacking (Harwood, 2023). Biolistic transformation offers a genotype-flexible alternative by bypassing the requirement for *Agrobacterium*-host compatibility and delivering DNA directly into cells, although it carries its own limitations, including complex integration patterns and higher cost. Particle bombardment provides an alternative delivery strategy that is largely independent of genotype, making it applicable to both model and non-model plant species while expanding vector delivery options. Successful application, however, requires careful optimization of physical and biological delivery parameters (Zalewski et al., 2012; Altpeter et al., 2016; Kapusi et al., 2017; Ozyigit and Kurtoglu, 2020; Gao, 2021; Thorpe et al., 2025). For example, biolistic transformation of tetraploid emmer wheat *Triticum dicoccum* (Schrank) achieved efficiencies of up to 12.9%, demonstrating its potential in genetically challenging backgrounds (Miroshnichenko et al., 2020).

Because transformation efficiency is tightly linked to regeneration capacity, CRISPR/Cas-mediated editing in barley remains largely restricted to a limited number of genotypes capable of reliably regenerating fertile plants from *in vitro* culture (Orman-Ligeza et al., 2020). Recent work has demonstrated that developmental regulators and transcription factors can partially overcome this limitation by enhancing regeneration in transformation-recalcitrant genotypes, including major cereal crops (Harwood, 2023).

For example, overexpression of the maize morphogenic regulators *BBM* and *WUS2* substantially increased transformation efficiency in otherwise recalcitrant lines of maize, indica rice, sugarcane, sorghum, and others (Lowe et al., 2016; Mookkan et al., 2017; Chaudhary et al., 2026). Similarly, expression of the wheat *GRF4-GIF1* chimera improved transformation efficiency across multiple species, including in wheat, triticale, rice, maize, citrus, strawberry, and other species (Debernardi et al., 2020; Feng et al., 2021; Biswal et al., 2023; Sanchez et al., 2024). More recently, co-expression of the barley morphogenic regulators *HvWUS* and *HvBBM2* was shown to enhance *Agrobacterium*-mediated transformation and regeneration in barley, including several genotypes with extremely low transformation capacity (He et al., 2025). Overexpression of *TaWOX5* has also been shown to enhance transformation and regeneration efficiency in cereals such as common wheat, einkorn wheat, triticale, rye, barley, and maize (Wang et al., 2022).

Efficient transformation coupled with robust regeneration is critical for successful application of advanced genome editing techniques, as gene knockout alone is often insufficient for breeding objectives (Li et al., 2024). Complex editing outcomes – including large deletions and inversions, multiplex target editing of several genes, and base or prime editing – typically occur at low frequency and therefore require generation of large populations of transformed plants. Obtaining numerous independent editing events increases the likelihood of identifying desirable genotypes while minimizing unintended effects such as off-target mutations or somaclonal variations (Kausch et al., 2019). These considerations highlight the need to develop rapid, reliable, and genotype-independent transformation and regeneration platforms in barley to support both direct improvement of commercial cultivars and fundamental research applications.

The objective of this study was to evaluate whether the wheat *GRF4–GIF1* morphogenic chimera, combined with optimization of key biolistic transformation parameters, including explant selection and plasmid DNA coating, can enhance transformation and regeneration efficiency in non-model, barley cultivars to support genome editing. The *GRF-GIF* system was selected because it has been shown to improve regeneration efficiency while minimizing the pleiotropic and developmental abnormalities often associated with *WOX* or *Wus*/*Bbm* overexpression (Lowe et al., 2016; Wang et al., 2022; Chaudhary et al., 2026). As a target, we edited the breeding-relevant barley *NUD* gene, which controls the naked/hulled grain phenotype and has previously been modified in the model cultivar Golden Promise (Gasparis et al., 2018; Gerasimova et al., 2020). To address this objective, we implemented a genotype-independent biolistic transformation workflow in three commercial barley cultivars, enabling recovery of stable transgenic and edited plants, including plasmid-free progeny, and demonstrating the feasibility of applying CRISPR/Cas9 editing directly in elite barley germplasm for precision breeding applications.

## 2 Materials and methods

### 2.1 Plant material and growth conditions.

Experiments were conducted using three high-yielding spring barley (*Hordeum vulgare* L.) cultivars: Tselinniy 5, Aley, and G-23035. These cultivars were sourced from the germplasm collection of the Siberian Research Institute of Plant Cultivation and Breeding branch of IC&G SB RAS. These cultivars were selected to represent commercially relevant spring barley germplasm adapted to Siberian agricultural conditions, with each possessing a distinct genetic background and varying regenerative capacities in immature embryo-derived tissues. Preliminary experiments aimed at optimizing biolistic transformation parameters were performed specifically using cv. Tselinniy 5, the cultivar with the lowest regeneration capacity among those tested, in order to develop parameters applicable to the most recalcitrant genotype before extending the workflow to additional cultivars. Although the tested cultivars exhibited varying regeneration capacities from immature embryo-derived tissues, repeated attempts in our laboratory to establish *Agrobacterium*-mediated transformation protocols for these cultivars were unsuccessful. These cultivars therefore provided an appropriate system for evaluating whether the *GRF4-GIF1* system could facilitate transformation of genotypes that are recalcitrant to conventional *Agrobacterium*-mediated transformation.

Donor plants were cultivated in a controlled-environment growth chamber (Fitotron SGR 121, Weisstechnik) at 15 °C/12 °C (day/night) under a 12-h photoperiod and 70% relative humidity for six weeks. At the tillering stage (BBCH 29/30), plants were supplemented with 15 g of Osmocote Exact Standart fertilizer (15% N, 9% P_2_O_5_, 12% K_2_O; Scotts, Netherlands). Subsequently, plants were transferred to a growth chamber maintained at 21 °C/18 °C (day/night) with a 16-h photoperiod (PPFD ≈ 440 µmol·m^−2^·s^−1^) and 70% humidity.

### 2.2 Construction of vectors for transformation and genome editing

Previously published guide RNA (gRNA) sequences, Nud45 (GGAGACCCAGGAGCCCCAG) and Nud50 (GCTCCTGGGTCTCCGAGATC), originally designed and validated in barley protoplasts by (Gerasimova et al., 2018), were used in this study. These gRNAs were subsequently applied for stable genome editing of the *NUD* gene in barley plants, and their high editing efficiency and off-target activity were characterized (Gerasimova et al., 2020). Although several alternative gRNA designs for this gene have been reported (Gasparis et al., 2018; Gerasimova et al., 2020; Hisano et al., 2025), earlier studies demonstrated that these specific guide sequences, Nud45 and Nud50, substantially outperform others in terms of on-target activity at the target locus, despite having confirmed off-target activity. Given the difficulties associated with the transformation of non-model cultivars, priority was given to maximizing editing efficiency at the expense of specificity.

The *NUD* gene sequences of the barley cultivars Tselinniy 5, Aley, and G-23035 were determined to verify their complementarity to the selected gRNAs. The target region was amplified using the PCR primers Hv_Nud_F1 and Hv_Nud_R2, previously described by Gerasimova et al. (2020), and the resulting PCR products were directly sequenced using the Sanger method.

**Figure 1:**
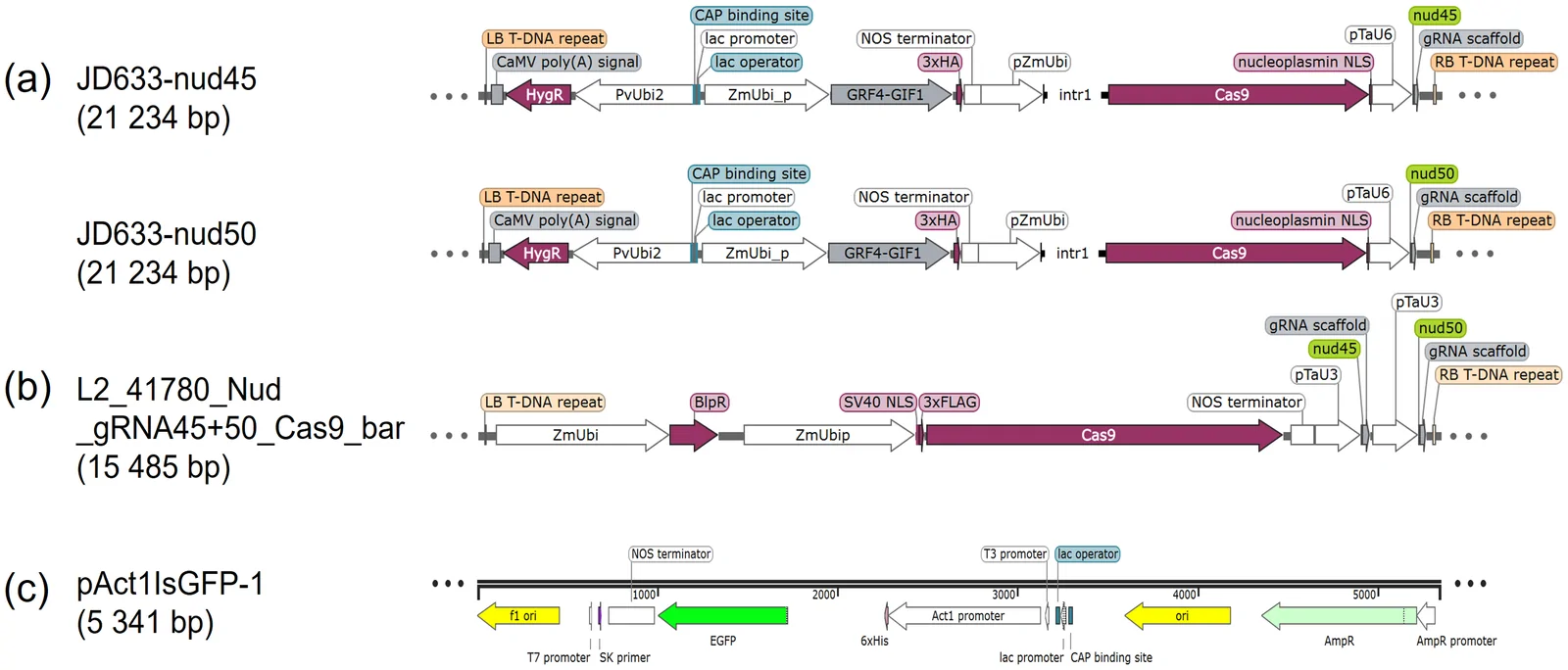
Schematic representation of the vectors used for CRISPR/Cas9-mediated editing. **(A)** JD633-nud45 and JD633-nud50. **(B)** L2_41780_Nud_gRNA45 + 50_Cas9_bar. **(C)** pAct1IsGFP-1.

Genome editing experiments were performed using the JD633 vector (Debernardi et al., 2020), which carries a wheat codon-optimized *Cas9* gene fused to a nuclear localization signal and driven by the maize *Ubiquitin* promoter (*pZmUbi*), a gRNA scaffold under the control of the wheat *U6* promoter (*pTaU6*), and the hygromycin resistance gene *hptII*. In addition, JD633 contains a *GRF4-GIF1* chimeric construct driven by (*pZmUbi*), which enhances regeneration capacity (**Figure 1**). The JD633 plasmid was a gift from Jorge Dubcovsky (Addgene plasmid #160393).

The gRNA sequences Nud45 and Nud50 (Gerasimova et al., 2020) (**Supplementary Table 1**) were cloned into the AarI sites of the JD633 vector. The resulting constructs were introduced into competent *E. coli* XL1-blue cells prepared using the Mix & Go kit (Zymo Research Cat. No. T3001). Plasmid DNA was isolated using the ZymoPURE II Plasmid Midiprep Kit (Zymo Research Cat. No. D4201). The final vectors, JD633-nud45 and JD633-nud50, were verified by Sanger sequencing.

To generate the L2_41780_Nud_gRNA45 + 50_Cas9_bar plasmid (L2) (**Figure 1**) for biolistic transformation of barley, which served as a control vector lacking growth factor genes, modules from the MoClo Kit – pFH85 (Addgene plasmid #128198), pFH33 (Addgene plasmid #125885), pICH47751 (Addgene plasmid #48002), pICH47761 (Addgene plasmid #48003), pFH66 (Addgene plasmid #131765), pFH114 (Addgene plasmid #128408), pICSL4723-P1 (Addgene plasmid #86173) – were assembled (Weber et al., 2011; Hahn et al., 2020). Assembly was performed using BsaI-HFv2 (NEB, USA) and BstV2I (SybEnzyme, Russia) restriction enzymes in combination with T4 DNA ligase, following published protocols (Hahn et al., 2020). The resulting L2_41780_Nud_gRNA45 + 50_Cas9_bar construct harbored two gRNAs with a classic sgRNA backbone for SpCas9, each driven by the wheat U3 promoter, a wheat codon-optimized SpCas9 gene under the control of the maize Ubi1 promoter, and the *bar* selectable marker gene (phosphinothricin acetyltransferase) driven by the *pZmUbi*. The final construct was validated by Sanger sequencing. Transformed tissues were selected on media supplemented with phosphinothricin (PPT).

Although the JD633 and L2 vectors contain the left and right T-DNA border sequences required for *Agrobacterium*-mediated transformation, these elements are not functional during particle bombardment and therefore do not influence DNA delivery.

It should be noted that different selectable marker systems were used for the JD633 (*hptII*, hygromycin) and L2 (*bar*, phosphinothricin) constructs, which represents a limitation of the study and may have influenced transformation outcomes. The choice of different selection systems was driven by the need to find an optimal balance between editing efficiency and the preservation of explant tissue viability in non-model cultivars. Given the generally higher sensitivity of elite barley cultivars to hygromycin and their lower regeneration capacity, the hygromycin concentration in experiments with JD633 was reduced to 35 mg/L (see section 2.4). In contrast, the L2 vector was utilized as a validated control providing a gentler selection pressure, similar to established protocols for barley and other cereals like wheat.

In addition, the pAct1IsGFP-1 vector was used as a reporter to monitor transformation efficiency. pAct1IsGFP-1 was a gift from Peggy Lemaux (Addgene plasmid #38775) (Cho et al., 2000).

### 2.3 Optimization of biolistic transformation parameters

#### 2.3.1 DNA coating of gold microparticles

To optimize biolistic transformation, the efficiency of two commercial lipofection reagents – TransIT-2020 (Mirus Bio, Madison, WI, USA) and Lipofectamine 3000 (Invitrogen, USA) – was compared with that of the conventional CaCl_2_/spermidine DNA-coating method. A plasmid mixture consisting of JD633-nud45 (450 ng), JD633-nud50 (450 ng), and pAct1IsGFP-1 (100 ng) was used for all treatments. In all cases, the total amount of plasmid DNA applied for coating was 1000 ng per shot.

For the CaCl_2_/spermidine method, plasmid DNA was coated onto gold particles according to the standard protocol described by (Sanford et al., 1993).

For DNA coating using TransIT-2020, plasmid DNA was added to washed 0.6 μm gold particles (Bio-Rad, USA; Cat. No. 1652262) under gentle vortexing, followed by the addition of the TransIT-2020 reagent (0.5 µL per shot) while continuing gentle vortexing. The mixture was incubated on ice for 10 min. The amount of TransIT-2020 was selected based on the protocol described by Miller et al. (2021). The mixture was then centrifuged at 8,000 × g for 30 s, the supernatant was discarded, and the resulting pellet was resuspended in nuclease-free water (10 µL per application) using brief sonication. The suspension was applied as small droplets onto macrocarriers positioned in their holder and allowed to air-dry for approximately 40 min.

DNA coating with Lipofectamine 3000 was performed following the same procedure as described for TransIT-2020, except that 0.3 µL Lipofectamine 3000 and 0.2 µL P3000 reagent were used per shot.

For each coating method, four biological replicates consisting of 15 explants each (60 explants in total) were bombarded. Transformation efficiency was assessed based on the frequencies of stable callus transformation and regeneration of transgenic plantlets.

#### 2.3.2 Evaluation of explant types for biolistic transformation

Two explant types from barley cv. Tselinniy 5 were evaluated for their transformation efficiency. Immature embryos (1.0 – 1.5 mm in length) were isolated and placed scutellum side up on barley callus induction medium (BCIM; Marthe et al., 2015). For the first experimental group, embryos were incubated overnight at 24 °C in the dark prior to bombardment. For the second group, embryos were cultured for 6–8 days to initiate immature embryo-derived callus (IE callus). In both groups, embryonic axes were excised one day before bombardment to prevent precocious germination.

The water-soluble cationic lipid TransIT-2020 was used as the DNA-coating agent, and a plasmid mixture consisting of JD633-nud45, JD633-nud50, and pAct1IsGFP-1 was applied in all treatments. For each explant type, four biological replicates, each consisting of 30 explants (120 explants per treatment), were bombarded. Transformation efficiency was assessed as the stable transformation frequency, defined as the number of independent transgenic events per 100 bombarded explants.

Detailed procedures for biolistic transformation, post-bombardment recovery, selection, and plant regeneration, optimized through preliminary experiments, are further described in the Sections 2.4 and 2.5.

### 2.4 Plant tissue culture

Barley spikes were harvested 12–15 days post-anthesis (dpa), when immature embryos reached approximately 1.0–1.5 mm in length. Seeds were surface sterilized by immersion in 70% (v/v) ethanol for 1 min, followed by incubation in a 50% commercial bleach solution (Domestos) for 10 min with gentle agitation. Surface-sterilized seeds were rinsed five times with sterile distilled water. Translucent immature embryos were isolated and placed scutellum side up on BCIM at a density of approximately 60 embryos per Petri dish for precultivation. To induce immature embryo-derived callus (IE-calli), explants were cultured at 24 °C in the dark for 6–8 days prior to bombardment.

The BCIM consisted of 4.3 g/L Murashige & Skoog (MS) basal salts, 30 g/L maltose·H_2_O, 1.0 g/L casein hydrolysate, 1.25 mg/L CuSO_4_·5H_2_O, 250 mg/L myo-inositol, 690 mg/L proline, 1.0 mg/L thiamine·HCl, 2.5 mg/L Dicamba, and 3.5 g/L Phytagel (pH 5.8).

One day before bombardment, embryonic axes were removed using sharp tweezers (Dumont, style 7, Cat. No. 0203-7-PO). Approximately 15 or 30 IE-calli were arranged within the central 2 cm diameter area of each dish. Four hours prior to and 16 hours following bombardment, IE-calli were maintained on osmotic medium (4.3 g/L MS salts with vitamins, 0.4 M mannitol, 30 g/L maltose, and 2.5 mg/L Dicamba) and incubated at 24 °C in the dark (Miroshnichenko et al., 2020).

Post-transformation culture, selection, and plant regeneration were performed according to (Marthe et al., 2015) with minor modifications. Specifically, Timentin was omitted from all media, as it is required only to suppress *Agrobacterium* growth following co-cultivation and is unnecessary in biolistic transformation. For the JD633 construct (*hptII* marker), the hygromycin concentration was reduced from 50 mg/L to 35 mg/L based on preliminary experiments in which IE-callus of the three cultivars were cultured on induction medium supplemented with hygromycin at 20, 30, 40, or 50 mg/L. At 50 and 40 mg/L, no callus induction was observed, whereas callus formed at 30 and 20 mg/L. The concentration of 35 mg/L was selected as providing sufficient selection pressure. For the L2 construct (*bar* marker), casein and L-glutamine were omitted from the culture medium, and phosphinothricin was used as the selective agent at concentrations established in published barley transformation protocols (Manoharan and Dahleen, 2002; Tobias et al., 2007). After bombardment, IE-callus (15 per plate) were transferred to BCIM supplemented with either 35 mg/L hygromycin (BCIM-Hyg) or 5 mg/L PPT (BCIM5P) and incubated at 24 °C in the dark for two weeks. Subculturing was performed every two weeks on fresh selection medium (35 mg/L hygromycin or 10 mg/L PPT for BCIM10P) for a total period not exceeding six weeks. Negative controls (one plate of 30 embryos per cultivar, bombarded without DNA) were included to verify selection stringency. No regenerants were recovered under hygromycin selection (JD633); under phosphinothricin selection (L2), regeneration was comparable to that of the experimental plates. Negative controls were not included in statistical analyses due to single-replicate design.

**Figure 2:**
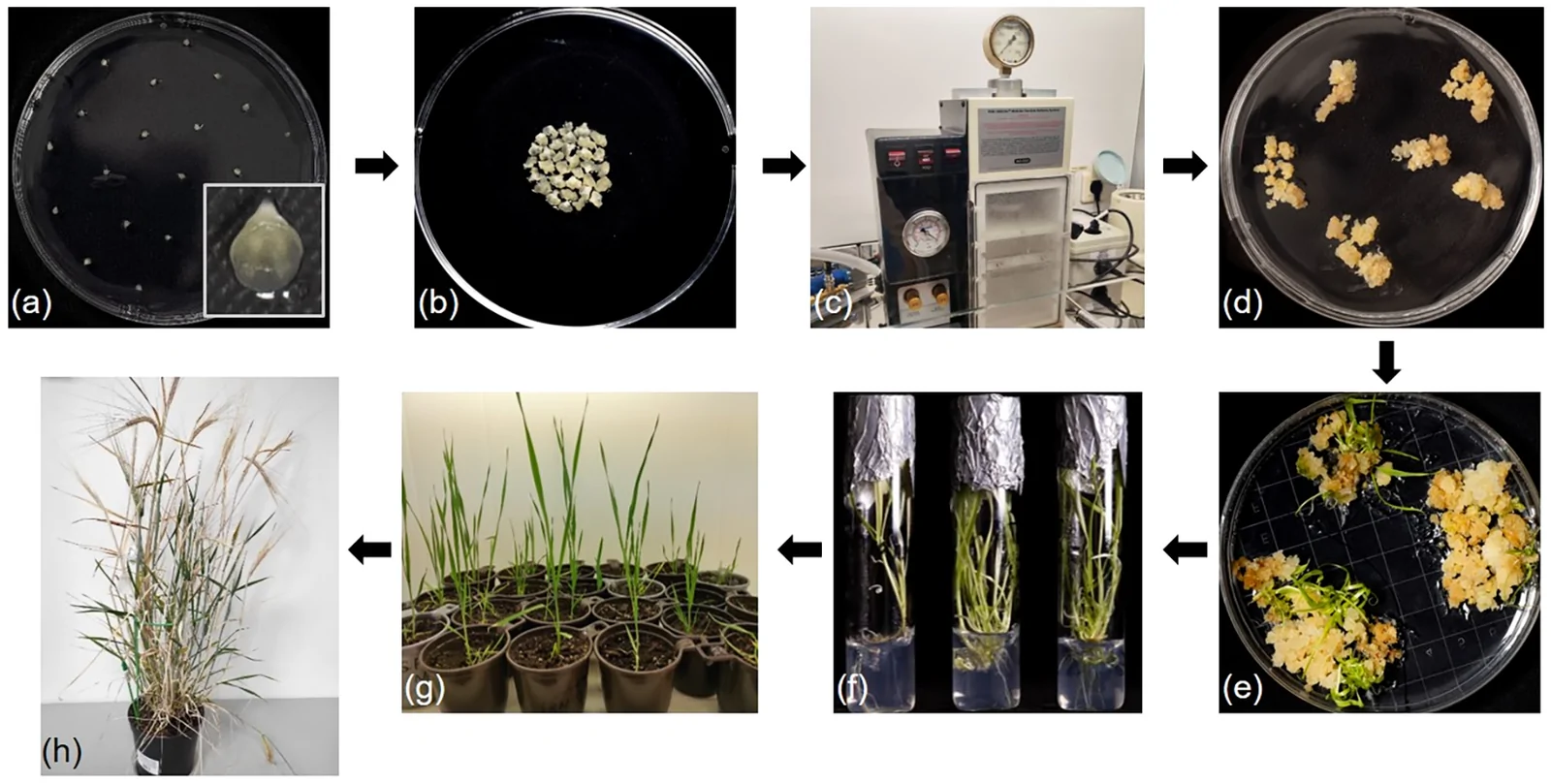
Visual overview of the *Hordeum vulgare* biolistic transformation pipeline: **(A)** Immature embryos on the day of isolation; the enlarged inset shows an embryo suitable for cultivation; **(B)** embryogenic callus placed on osmoticum medium for 4 h and arranged ready for biolistic; **(C)** Particle bombardment using the gene gun; **(D)** enlarged embryogenic callus developing from an immature embryo after 4–5 weeks of selection; **(E)** the initiation of shoots on regeneration medium; **(F)** transgenic barley rooting in glass culture tubes; **(G)** transgenic barley plantlets after transfer to soil; **(H)** transgenic barley growing to maturity in the controlled-environment chamber.

Following selection, yellowish embryogenic calli were transferred to barley regeneration medium (BRM) supplemented with either 15 mg/L hygromycin (BRM-Hyg) or 5 mg/L PPT (BRM5P). Cultures were maintained at 24 °C under fluorescent light (~100 μmol m^−2^ s^−1^) with a 12-h photoperiod for 2–4 weeks. The BRM (pH 5.8) contained K4N macronutrients, K micronutrients, 36 g/L maltose, 27.5 mg/L NaFeEDTA, B5 vitamins, 1.25 mg/L CuSO_4_·5H_2_O, 0.225 mg/L 6-Benzylaminopurine (6-BAP), and 3.5 g/L Phytagel. The salt compositions were based on the formulations originally described by (Kumlehn et al., 2006), while the final media concentrations were utilized as modified by Marthe et al. (2015) (Marthe et al., 2015). Regenerants with developed shoots and roots were transferred to hormone-free BRM in plant culture containers (Phytohealth, SPL) or individual glass tubes until robust root systems formed. T_0_ plantlets were then transplanted into soil and grown to maturity to obtain T_1_ seeds. Representative stages of the tissue culture process are shown in **Figure 2**.

### 2.5 Transformation via particle bombardment

Particle bombardment was performed using a PDS-1000/He Biolistic Particle Delivery System (Bio-Rad, USA). For every ten shots, 50 µL of washed 0.6 µm gold particles (40 mg/mL; Bio-Rad, USA, Cat. No. 1652262) were coated with a DNA mixture consisting of 9000 ng of plasmid DNA, comprising either the JD633-nud45/JD633-nud50 vectors or the L2_41780_Nud_gRNA45 + 50_Cas9_bar vector, supplemented with 1000 ng pAct1IsGFP-1 as a visual reporter. DNA precipitation was mediated by TransIT-2020 (0.5 µL per shot; Mirus Bio, USA). The procedure for DNA coating onto gold particles using TransIT-2020 is described in the Section 2.3.1.

The bombardment parameters were selected based on our previous experience with biolistic transformation of cereals, including wheat (Kiseleva et al., 2025), and were maintained constant throughout the present study. Immature barley calli were bombarded at a target distance of 6.0 cm from the stopping screen using 1100 psi rupture disks (Bio-Rad, USA, Cat. No. 1652328) and a vacuum of approximately 28” Hg. For each cultivar, four biological replicates, each consisting of 30 calli (120 calli per cultivar) were bombarded.

### 2.6 Fluorescent visualization

GFP fluorescence was used only as a qualitative criterion for successful transformation for every plate, as the heterogeneous surface and multilayered structure of embryogenic callus precluded reliable quantitative assessment. Fluorescence imaging of immature embryos and IE-calli was performed using a ZEISS SteREO Discovery.V8 stereomicroscope (Carl Zeiss MicroImaging GmbH, Jena, Germany) equipped with a filter cube 38 for GFP detection (excitation: BP 470/40; beam splitter: FT 495; emission: BP 525/50). Images were captured using an AxioCam 280 color CCD camera and processed with ZEN 3.1 software (Carl Zeiss MicroImaging GmbH). Fluorescence screening was conducted at a magnification of 1.6× with microscope objective and 10× eyepieces and a reflection magnification of 1.

### 2.7 Identification of mutations and vector integration

To identify vector integration and targeted mutations, genomic DNA was extracted from calli, leaves of regenerated T_0_ plantlets, individual T_0_ shoots at the heading stage, and T_1_ plants. Plasmid integration was confirmed by PCR using primers specific to the *hptII*, *bar*, and gRNA sequences (**Supplementary Table 1**).

The presence of mutations in the *NUD* gene and potential off-target sites was assessed by PCR screening followed by Sanger sequencing and next-generation sequencing (NGS).

To detect both short and long insertions/deletions (indels) within the target gene, PCR primers previously described by Gerasimova et al. (2020) were utilized (**Supplementary Table 1**). Based on previous reports, the Nud45 gRNA has three additional genomic targets flanked by a PAM, while the Nud50 gRNA has three predicted off-target sites lacking a PAM. Primers for identifying potential off-targets were adopted from Gerasimova et al. (2020) (**Supplementary Table 1**), and additional primers for the 6H off-target locus were designed in this study.

Large indels were identified by PCR product size analysis on agarose gels, and the resulting amplicons were purified from 2% agarose gels using the Zymoclean Gel DNA Recovery Kit (Zymo Research, USA). Purified products were sequenced using the BigDye Terminator v.3.1 Cycle Sequencing Kit (Applied Biosystems, Foster City, CA, USA). Sequencing reactions were purified with the ZR DNA Sequencing Clean-Up Kit (Zymo Research, USA) and analyzed on an ABI 3130XL Genetic Analyzer at the SB RAS Genomics Core Facility.

To identify the spectrum of possible mutations in the calli, Sanger sequencing of amplicons of the target genes was performed. The chromatograms were subsequently analyzed using DECODR 3.0 (Bloh et al., 2021). As a reference chromatogram, a file obtained from amplicons of control calli (subjected to biolistic treatment without the use of a vector) was used. The *r^2^* values, which measure goodness of fit, averaged 0.98, indicating high reliability of the deconvolution results.

For NGS analysis, amplicons corresponding to the target and off-target genes (**Supplementary Table 1**) were purified using CleanMag DNA magnetic beads (Evrogen, Russia). Libraries were sequenced on an Illumina MiSeq platform, generating paired-end 250-bp reads. Sequence alignment and mutation quantification were performed using CRISPResso2 (https://github.com/pinellolab/CRISPResso2) (Clement et al., 2019). Indel frequencies were calculated in Cas9 mode using default parameters, with a minimum alignment homology threshold of 90% and an editing window of ±1 bp around the predicted cleavage site. Mutations were classified as heterozygous when modified and unmodified sequences each accounted for approximately 50% of the total reads. NGS results were further validated by Sanger sequencing, with data analysis performed using the DECODR 3.0 (Bloh et al., 2021). As a reference chromatograms, a files obtained from amplicons of the intact cultivars were used. The *r^2^* values, which measure goodness of fit, averaged 0.98.

### 2.8 Transgene copy number estimation

To estimate plasmid insertion copy number, quantitative PCR (qPCR) was performed using a relative quantification approach. The relative abundance of *hptII* and/or gRNA-specific amplicons (**Supplementary Table 1**) was normalized to the endogenous single-copy barley gene *CONSTANS2* (*HvCO2*). All reactions were conducted in three replicates for each plant.

qPCR reactions contained 30 ng of genomic DNA in a total volume of 20 µL using PowerUp™ SYBR Green Master Mix and were run on a QuantStudio™ 5 Real-Time PCR System (Thermo Fisher Scientific, Vilnius, Lithuania). Individual PCR efficiency (E) values were determined using LinRegPCR software (Ruijter et al., 2014). Relative transgene copy number was calculated according to the Pfaffl method (Pfaffl, 2001), using the sample with the highest Cq value as a calibrator.

Quantitative PCR-based approaches using normalization to a single-copy reference gene are widely applied for estimation of transgene copy number in plants (Ingham et al., 2001; Bubner and Baldwin, 2004).

### 2.9 Statistical analysis

Transformation and genome editing efficiencies were evaluated based on the molecular analysis of all recovered regenerants. Stable transformation frequency was defined as the number of independent transgenic events per 100 bombarded explants. Because particle bombardment may generate multiple independent transformation events within a single scutellum-derived callus, all regenerated plantlets were analyzed individually. Regenerants originating from independent explants were considered independent transformation events. Regenerants originating from the same callus fragment and carrying identical mutation profiles were considered clonally derived and were therefore counted as a single transformation event. In contrast, regenerants originating from the same callus exhibiting distinct mutation profiles at the *NUD* target locus and three off-target loci were regarded as independent transformation events. Genome editing efficiency (mutagenesis frequency) was determined as the percentage of independent plants carrying mutations within the target sequences relative to the total number of confirmed transgenic lines.

Statistical analyses were performed using the R (version 4.3.0) and using the *rstatix* package (version 0.7.2) (Kassambara, 2025). Data were tested for normality and homogeneity of variance using the Shapiro-Wilk and Levene’s tests prior to analysis. Transformation efficiency and regeneration frequency were calculated independently for each of the three or four biological replicates (each replicate included 30 explants). Because these variables were expressed as percentages and the number of biological replicates was limited, no assumption of normality was made. Therefore, differences among experimental groups were assessed using the non-parametric Kruskal-Wallis test followed by Dunn’s multiple comparison test. A p-value < 0.05 was considered statistically significant. All experiments included at least three biological replicates.

## 3 Results

### 3.1 Optimization of biolistic transformation parameters

Although biolistic delivery is an effective method for introducing DNA, RNA, or ribonucleoprotein (RNP) complexes into plant cells, its efficiency and reproducibility can vary significantly depending on experimental parameters (Altpeter et al., 2016). We therefore evaluated how two factors – the reagent used for DNA coating onto gold microcarriers and type of tissue used as explant material – affect transformation efficiency.

We emphasize that optimization of these parameters is particularly important for non-model barley cultivars, for which existing biolistic protocols are generally less efficient in producing stable transformation events compared to Golden Promise barley and other well-characterized genotypes (Zalewski et al., 2012; Kapusi et al., 2017).

In this study, we focused on two critical factors: (i) the chemical reagents used for coating gold particles with vector DNA, and (ii) the type and physiological state of the explant tissue. We hypothesized that both the stability of the DNA–gold complex and the physiological condition of the explant are key determinants influencing transformation efficiency, affecting both DNA delivery and tissue viability.

#### 3.1.1 Comparison of two types of explants for the biolistic transformation

The choice of starting plant material is a critical factor influencing transformation success in crop species. In barley, highly efficient *Agrobacterium*-mediated transformation systems using immature embryos as explants has been established; however, these systems are largely restricted to the cultivar Golden Promise.

**Figure 3:**
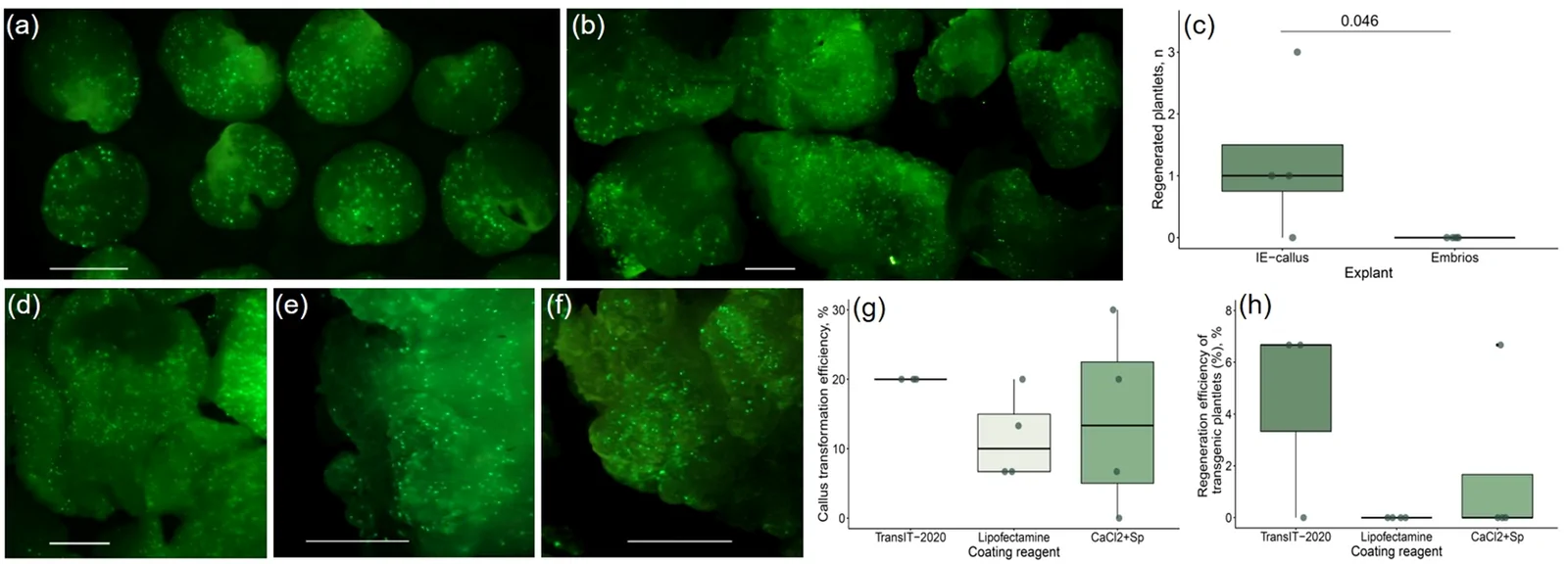
Optimization of biolistic transformation parameters in barley cultivar Tselinniy 5. **(A, B)** Transient GFP expression in immature embryos **(A)** and immature embryo-derived callus **(B)** on the second day post-bombardment. Scale bar = 1 mm.; **(C)** Boxplot showing the number of regenerated plantlets obtained after biolistic transformation of two explant types (IE callus and embryos) using a mixture of the JD633-nud45 and JD633-nud50 vectors. Results from biological replicates (n = 4 per explant type) are indicated by green points. Each replicate included 30 explants. Statistical significance between groups is shown above the boxes (p-values), calculated using the Kruskal–Wallis test followed by Dunn’s *post hoc* test.; **(D–F)** Transient GFP expression in IE callus two days post-bombardment using different DNA-coating reagents: **(D)** TransIT-2020-mediated DNA delivery, **(E)** Lipofectamine 3000-mediated delivery, and **(F)** conventional CaCl_2_/spermidine precipitation. Scale bar = 1 mm.; **(g, h)** Boxplots showing callus transformation efficiency **(a)** and regeneration efficiency of transgenic plantlets **(b)** using different DNA-coating reagents. Results from biological replicates (n = 3 for TransIT-2020 and n = 4 for Lipofectamine 3000 and CaCl_2_/spermidine) are indicated by green points. Each replicate included 15 explants.

In this study, we compared two explant types: immature embryos (approximately 1.0-1.5 mm in size), which are widely used for crop transformation, and immature embryo-derived callus (IE-callus), which is less commonly employed for this purpose (Bekalu et al., 2023). Explants were bombarded with a mixture of the JD633 vector and the pAct1IsGFP-1 reporter construct. Transient GFP expression was detected in both immature embryos and IE calli one day after bombardment and became more pronounced after two days. Both explant types exhibited numerous fluorescent foci (**Figure 3**).

After four weeks of cultivation on BCIM-Hyg medium, callus formation was observed from both explant types. Two types of callus were observed during the culture process: compact, globular embryogenic callus, which was capable of shoot regeneration, and friable, translucent non-embryogenic callus that failed to differentiate. A preliminary analysis was conducted to verify CRISPR/Cas9 activity. Genomic DNA was extracted from randomly selected calli maintained under selection (four per explant type), and the target *NUD* locus was analyzed by Sanger sequencing. Targeted mutations were detected in both explant types (**Supplementary Figure 1**). The proportion of sequences containing mutations was approximately 10–11% in both cases. In IE calli, four mutant alleles were identified, including a 48-bp deletion. In immature embryos, three mutant alleles were detected, consisting of single- or three-nucleotide deletions and a 1-bp insertion.

Significant differences were observed in the capacity for plant regeneration (p-value < 0.05). Following transformation and subsequent culture, no regenerated plants were recovered from immature embryos, whereas five plants were obtained from independent IE calli (**Table 1**; **Supplementary Table 2**). Among the regenerated plantlets, three were confirmed to contain the vector integration. These results indicate that explant type strongly influenced regeneration capacity and, consequently, stable transformation efficiency. IE callus demonstrated superior performance in generating stable transformants and was therefore selected for subsequent experiments.

**Table 1:** Efficiency of generating stable T_0_ transgenic plants following biolistic transformation of immature barley embryonic tissues from the cultivar Tselinniy 5 using the JD633 vector.

Type of explants	No. of explants^*^	No. of regenerated plantlets	No. of PCR- positive plants and efficiency ^**^
immature embryos	120	0	0 (0%)
embryo-derived callus	120	5	3 (2.5%)

* 30 explants for every tissue type were bombarded and cultivated per culture dish, with four dishes being used as repetitions per treatment.

** PCR was conducted using primers specifically amplifying the *hptII*, Nud45 and Nud50 transgene, and the transformation efficiency was calculated as the number of PCR-positive plants per number of bombarded explants.

#### 3.1.2 Evaluation of DNA coating reagents on transformation frequency

High densities of transient fluorescence foci were observed across all three DNA coating methods (**Figures 3D–F**), indicating successful plasmid DNA delivery. However, when using the conventional CaCl_2_/spermidine precipitation protocol, the density of GFP transient expression foci varied more substantially among biological replicates compared with TransIT-2020-mediated delivery.

The coating methods differed in their effects on subsequent tissue recovery and stable transformation. Following cultivation on selective media, TransIT-2020 yielded the highest frequency of viable embryogenic calli (20.0%, 9 out of 45 explants). Lipofectamine 3000 and the conventional CaCl_2_/spermidine method produced frequencies of 11.7% and 14.2% (7 and 9 of 60 explants per treatment), respectively (**Table 2**; **Supplementary Table 3**). Genomic DNA was extracted from a subset of surviving calli, and vector integration was confirmed by PCR using specific primers.

**Table 2:** Comparative efficiency of different DNA coating reagents on callus induction and stable plant regeneration in barley (cv. Tselinniy 5).

Vector	Coating DNA reagent	No. of IECs^**^	No. of recovered stable embryogenic calli (average frequency %)	No. of PCR- positive plants and efficiency ^***^
JD633_nud 45/50	TransIT-2020	45*	9 (20%)	2 (4.4%)
JD633_nud 45/50	Lipofectamine	60	7 (11.7%)	0 (0%)
JD633_nud 45/50	CaCl_2_+Sp	60	9 (14,2%)	1 (1.7%)

* one repetition was lost because of contamination.

** 15 immature zygotic embryo-derived calli (IECs) for every coating DNA reagent were bombarded and cultivated per culture dish, with four dishes being used as repetitions per treatment.

*** PCR was conducted using primers specifically amplifying the *hptII*, Nud45 and Nud 50 transgene integration, and the transformation efficiency was calculated as positive callus or plants per number of bombarded explants.

Differences among treatments became more evident during the regeneration stage. Two stable T_0_ plantlets were recovered following TransIT-2020 treatment, whereas only one plantlet was obtained from the CaCl_2_/spermidine group.

No stable regenerants were recovered after Lipofectamine 3000 treatment. Due to limited sample size and low plant regeneration frequency, the observed differences did not reach statistical significance. Larger experimental sample sizes will be required to confirm these trends. Under the conditions tested, TransIT-2020 provided the most consistent performance for DNA coating during transformation of barley IE-calli.

The conventional CaCl_2_/spermidine method is known to be sensitive to ambient humidity and can result in variable DNA precipitation and microcarrier aggregation, potentially increasing cellular damage (Miller et al., 2021). In contrast, TransIT-2020 protocol provided more consistent coating performance under the conditions tested. Based on its higher recovery of stable T0 regenerants and operational reproducibility, TransIT-2020 was selected for subsequent experiments.

### 3.2 Impact of the *GRF4-GIF1* chimera on transformation efficiency and plant regeneration

Following preliminary optimizations, we evaluated the efficiency of the JD633-nud45/50 vectors incorporating the *GRF4-GIF1* chimera in three commercial spring barley cultivars: Tselinniy 5, Aley, and G-23035. The L2_41780_Nud_gRNA45 + 50_Cas9_bar (L2) vector, previously shown to be effective in wheat (Kiseleva et al., 2025), was used as a control to assess the contribution of morphogenic regulators to transformation and genome editing outcomes. In both experiments, the pAct1IsGFP-1 reporter plasmid was co-delivered.

**Figure 4:**
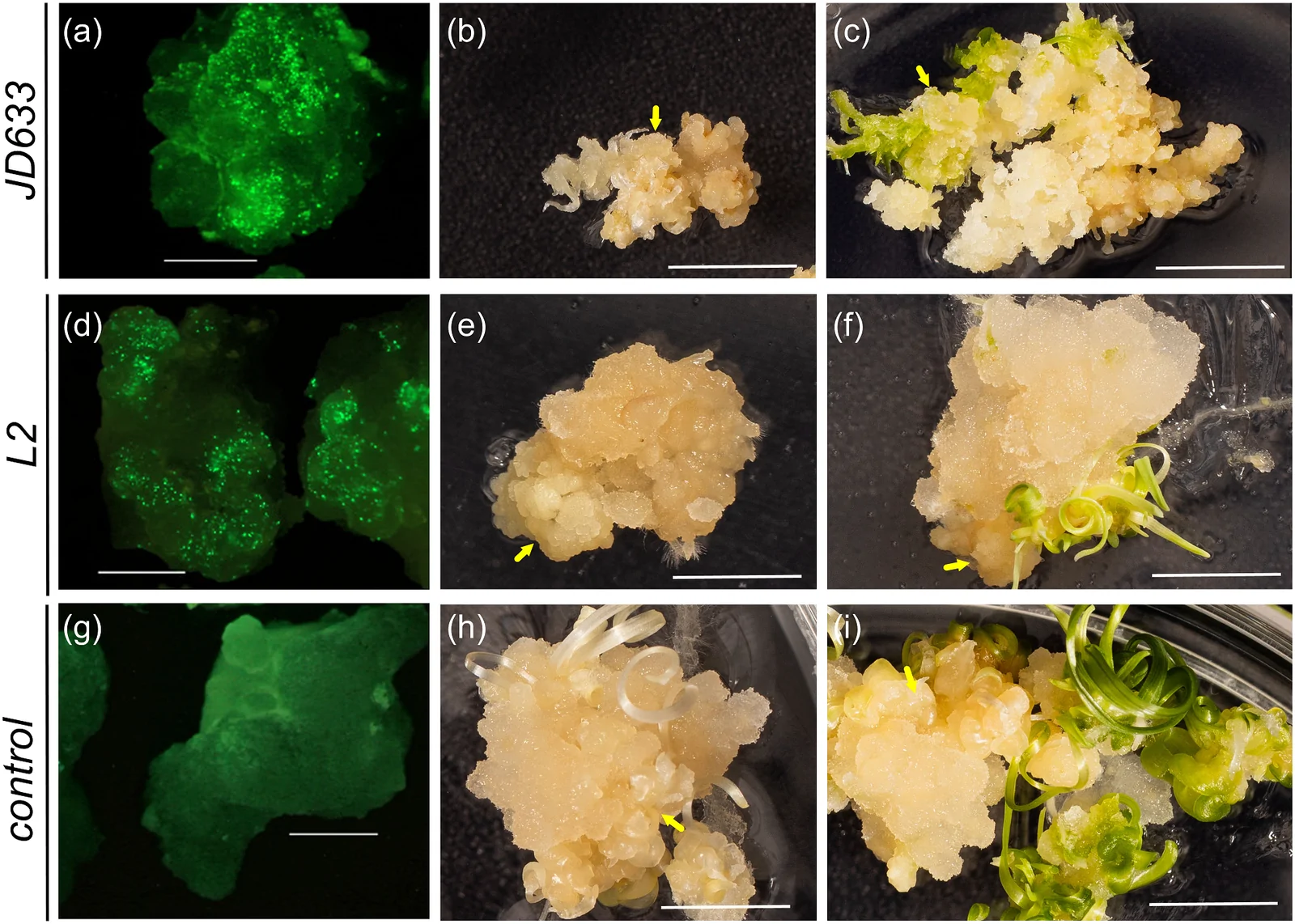
Comparison of tissue culture stages for calli transformed with two different plasmids versus non-transformed controls (lower row). **(A, D)** Transient GFP expression in immature embryo-derived calli two days after biolistic transformation **(G)** control non-transformed callus. **(B, E, H)** Calli appearance after two cycles of induction (28 days) on induction medium (with selection for transformed lines and without selection for control tissues) for vectors JD633 and L2. **(C, F, I)** Initiation of shoot regeneration after two weeks on regeneration medium. Scale bar **(A, D, G)** = 1 mm; **(B, C, E, F, H, I)** = 1сm. Arrows indicate embryogenic callus characterized by yellowish-white color and a compact globular structure.

Different selective agents were applied according to vector-specific marker genes (*bar* for L2 and *hptII* for JD633), both of which have been previously reported as suitable for barley transformation (Wan and Lemaux, 1994; Zang et al., 2022). Under the conditions tested, differences in selection stringency were observed between the two systems. Phosphinothricin (PPT) selection in the L2 control group permitted extensive proliferation of embryogenic calli across all cultivars (**Figures 4E, F**). This resulted in the regeneration of 62 plantlets, all of which were later identified as non-transgenic escapes (**Table 3**; **Supplementary Table 4**). In contrast, IE-calli transformed with the *GRF4-GIF1*-containing JD633 vector and selected on hygromycin developed a compact morphology after 5–6 weeks of cultivation on BCIM-Hyg (**Figure 4B**; **Supplementary Figure 2**) As a result of this treatment, 27 regenerated seedlings of all three varieties were obtained in total.

**Table 3:** Efficiency of genetic transformation and targeted mutagenesis in T0 seedlings of barley.

Variety	Vector	No. of IECs^*^	No. of regenerated plantlets	Number of independently transformed plants and efficiency ^**^	No. of independent edited plants and efficiency ^***^
Tselinniy 5	JD633	120	5	3 (2.5%)	3 (2.5%)
Aley	JD633	120	9	5 (4.2%)	4 (3.3%)
G-23035	JD633	120	13	6 (5%)	2 (1.7%)
Tselinniy 5	L2	120	10	0 (0%)	0 (0%)
Aley	L2	120	36	0 (0%)	0 (0%)
G-23026	L2	120	16	0 (0%)	0 (0%)

* 30 immature zygotic embryo-derived calli (IECs) for every cultivar were bombarded and cultivated per culture dish, with four dishes being used as repetitions per treatment.

** PCR was conducted using primers specifically amplifying the *hptII* or *bar*, Nud45 and Nud 50 transgene insertion, and the transformation efficiency was calculated as number of independent events per number of bombarded explants.

*** editing efficiency was calculated as number of independent mutant plants per number of bombarded explants; the frequency of editing among recovered transgenic lines reached up to 64,3%.

Callus morphology differed between treatments. During induction, *GRF4-GIF1*-transformed calli were smaller than control calli. After two weeks on the regeneration medium (BRM-Hyg), their size became comparable to or exceeded that of the controls. Additionally, *GRF4-GIF1*-transformed calli exhibited elongated, filamentous outgrowths and a fibrous structure that were not observed in the control group (**Figure 4B, C**; **Supplementary Figure 2**). Despite these transient tissue characteristics, plants regenerated from *GRF4-GIF1*-treated calli displayed normal development without detectable pleiotropic abnormalities. Thus, the morphological alterations associated with *GRF4-GIF1* expression are confined to the callus stage and do not translate into developmental abnormalities in regenerated plants.

Although the total number of regenerants was 27 plantlets, PCR analysis revealed that 15 of them contained stable vector integration. Previous studies have shown that different cell lineages within a single embryogenic callus may undergo independent double-strand break (DSB) repairs, resulting in regenerants with unique mutational profiles derived from the same explant (Zang et al., 2022). To accurately determine transformation efficiency, we discriminated between independent genetic events and clonal regenerants originating from the same callus. The recovery of multiple independent events enabled the identification of edited plants free from the off-target effects or somaclonal abnormalities observed in some of the other lines.

Analysis of mutation profiles and callus origin revealed that several plants were clonal. Specifically, for cultivar G-23035, the plants G-303 and G-321 shared identical mutation profiles and were treated as a single event. In contrast, plants A-315 and A-388 (cultivar Aley), although derived from the same callus, carried distinct mutations and were thus considered independent genetic events based on their distinct mutation profiles across multiple loci. Similarly, plants T-C8, T-D8, and T-331 (cultivar Tselinniy 5) originated from a single callus and were treated as a single genetic event (see Section 3.4 for details). Consequently, the 15 PCR-positive plants represented 12 independent stable transformation events. Additionally, two plants lacking vector integration carried targeted mutations. Thus, a total of 14 independent genetically modified events were identified (**Supplementary Table 5**). In the absence of the *GRF4-GIF1* system, no transgenic plants were recovered from any of the three cultivars tested (despite bombardment of 120 explants per cultivar).

To ensure accuracy, transformation and editing frequencies reported in **Table 3** were calculated based on independent transformation events. Regenerants originating from the same callus fragment and exhibiting identical mutation profiles (e.g., G-303/G-321 and T-C8/T-D8) were treated as a single event (**Table 3**; **Supplementary Table 4**).

Plasmid integration in regenerated plants was identified by PCR using primers specific to the *hptII* (Hyg), *Bar* and gRNA sequences (**Supplementary Table 1**). A detailed summary of molecular screening results is provided in **Supplementary Table 5**.

Introduction of the JD633 vector carrying the *GRF4-GIF1* chimera significantly increased transformation efficiencies of 5.0% for G-23035, 4.2% for Aley, and 2.5% for Tselinniy 5. Statistical analysis using the Kruskal-Wallis/Dunn test confirmed that, when compared to the L2 control, JD633 significantly increased the recovery of independent edited plants in Tselinniy 5 (p = 0.04) and Aley (p = 0.04), whereas the effect in G-23035 was not statistically significant. A similar trend was observed for editing efficiency, with significant improvements in Tselinniy 5 (p = 0.04) and Aley (p = 0.04), but not in G-23035 (**Supplementary Table 4.2**). For independent transgenic plant recovery, significant differences between JD633 and L2 were detected in Aley (p = 0.008) and G-23035 (p = 0.01), while no significant effect was observed in Tselinniy 5 (**Supplementary Table 4.2**). The same pattern was observed for transgenic plant efficiency, confirming a genotype-dependent response to the morphogenic construct.

When data were averaged across all cultivars, JD633 showed a highly significant overall improvement over L2 in all evaluated parameters, including independent edited plants (p = 0.0007), editing efficiency (p = 0.0007), independent transgenic plants (p = 0.0002), and transgenic plant efficiency (p = 0.0002) (**Supplementary Table 4.3**).

These findings indicate that inclusion of the *GRF4-GIF1* module, together with hygromycin selection, improves recovery of stable transformants in the tested barley cultivars.

### 3.3 Targeted editing of the *NUD* gene

The *NUD* gene was selected as a genome editing target because its loss-of-function phenotype is readily distinguishable: frameshift mutations result in naked (hull-less) grains instead of the wild-type hulled phenotype (Taketa et al., 2008; Gasparis et al., 2018). Any frameshift within the coding sequence disrupts *NUD* function and leads to conversion from hulled to naked barley. Two previously published gRNA sequences, Nud45 (GGAGACCCAGGAGCCCCAG) and Nud50 (GCTCCTGGGTCTCCGAGATC), reported to efficiently disrupt *NUD*, were used to target conserved regions within the first exon.

Because transient expression of CRISPR/Cas9 vectors can generate mutations without stable DNA integration (Zhang et al., 2016; Danilo et al., 2019; Qiu et al., 2022; Kiseleva et al., 2025), all 89 regenerated plants were screened for genomic edits regardless of vector integration status. Target regions of the *NUD* gene were analyzed by Sanger sequencing and Next-Generation Sequencing (NGS). No mutations were detected among the 62 plants regenerated using the L2 control vector, whereas edited plants were identified among plants regenerated with the JD633 vectors (**Table 3**; **Supplementary Tables 4, 5**).

**Figure 5:**
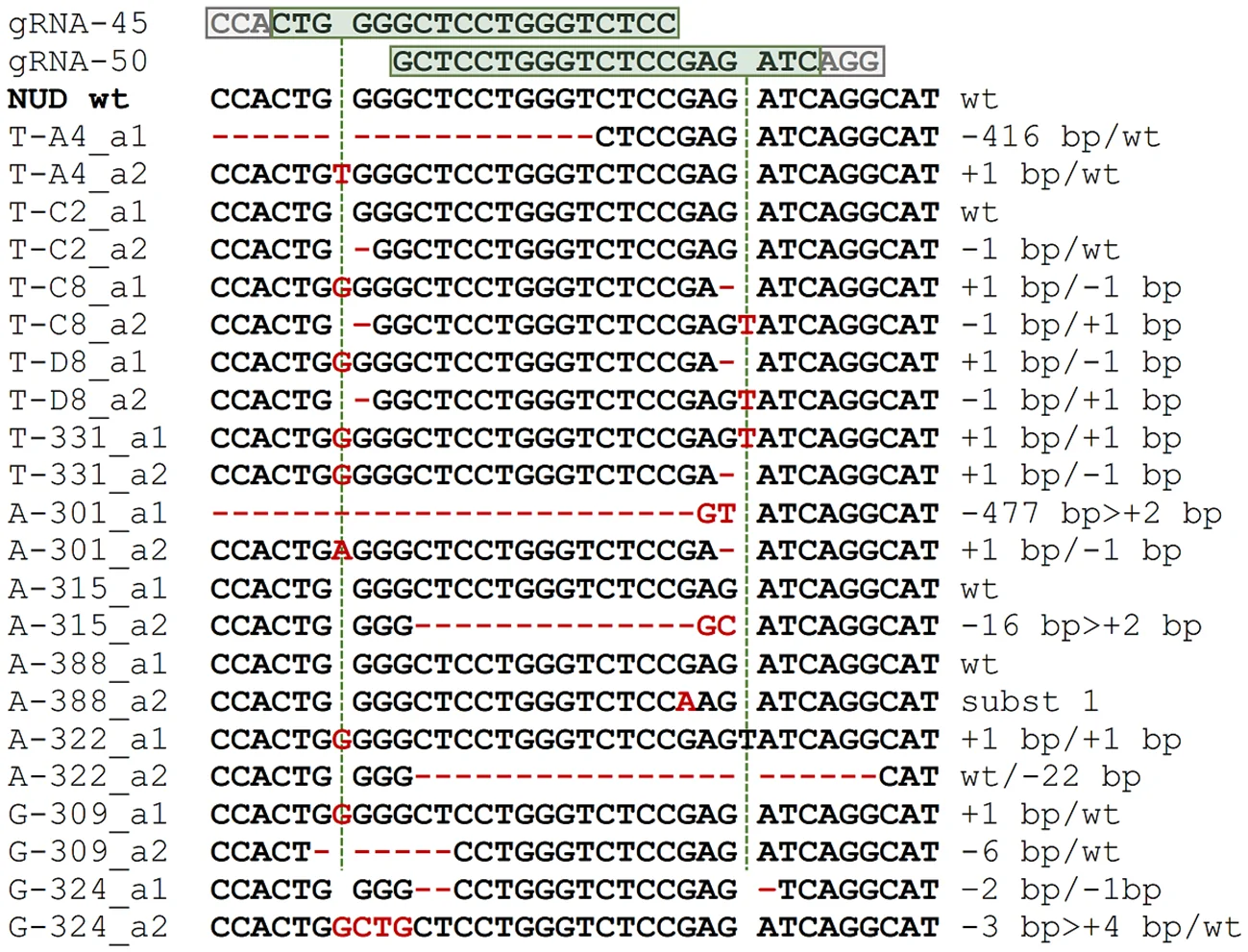
Sequences of the *NUD* gene in T0 plants regenerated after transformation with JD633 carrying gRNAs Nud-45 and Nud-50. Sequences in green box indicate the gRNA target sites, sequences in grey box indicate PAM motif. Vertical dashed lines mark the predicted Cas9 cleavage sites. If a plant carried two or more distinct alleles, they were designated as a1 and a2 following the plant name. Mutations in the sequence are colored in red.

Among plants regenerated following JD633-mediated transformation (*GRF4-GIF1*), 11 of 27 total regenerated plants carried mutations at the target site (**Supplementary Table 5**). Nine plants contained both vector integration and mutations, whereas five carried vector sequences without detectable modifications of *NUD*. Two plans exhibited mutations in the *NUD* gene in the absence of detectable plasmid integration, suggesting that these mutations resulted from transient CRISPR/Cas9 expression. In total, five mutants (three independent events) were obtained for Tselinniy 5, four for Aley, and two for G-23035. The overall editing efficiency ranged from 1.7% to 3.3% based on the total number of treated explants. Simultaneously, among the successfully transformed lines, the editing frequency was as high as 64.3%. Sequence analysis revealed that the most frequent mutations were small insertions/deletions (1–2 bp), although deletions ranging from 3 to 22 bp were also observed (**Supplementary Table 5**; **Figure 5**). Two plants carried large deletions of 416 bp (T-A4) and 477 bp (A-301) in *NUD* genes (**Figure 5**).

**Figure 6:**
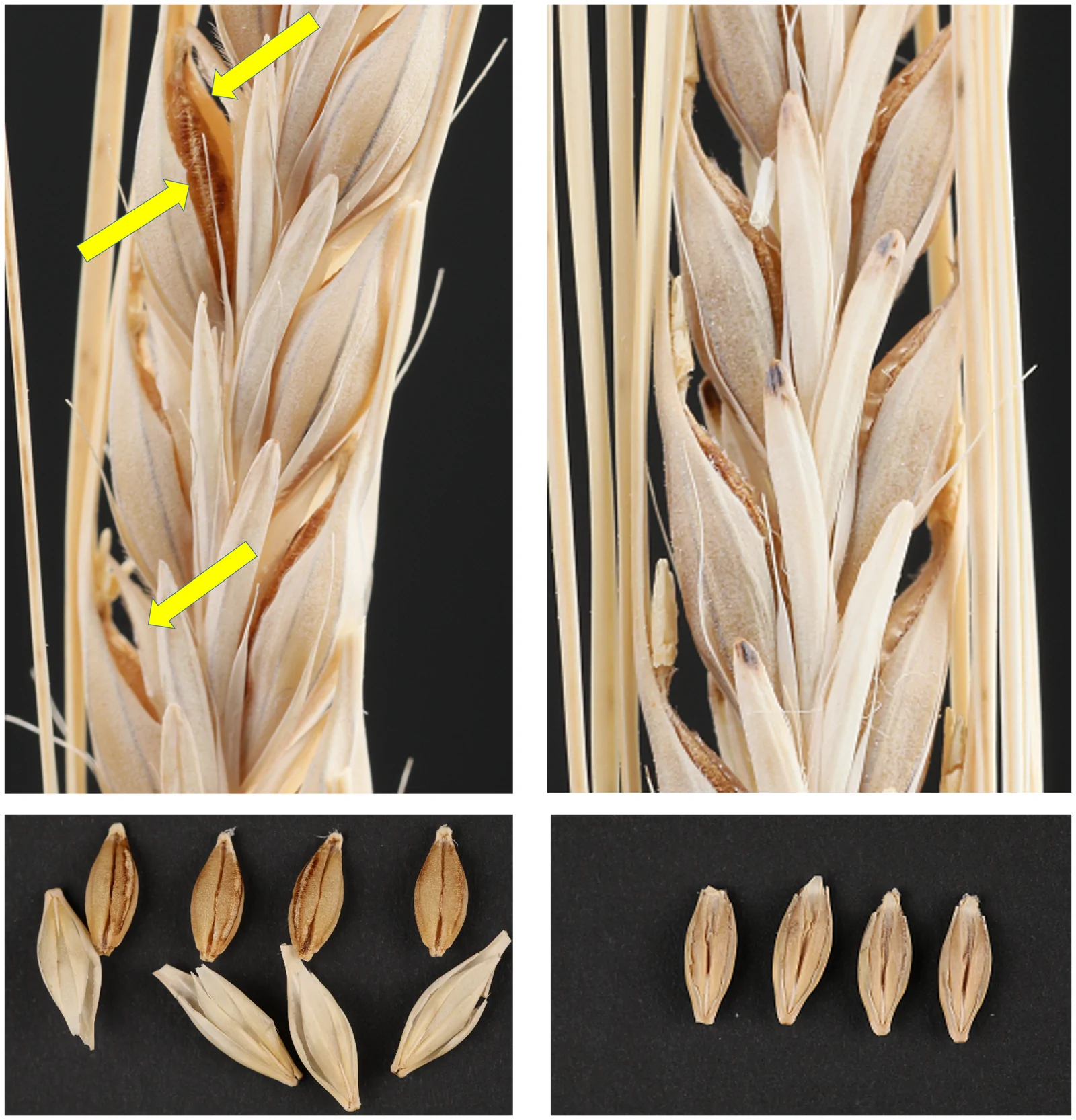
Phenotypic characterization of the *NUD* gene knockout in barley. Upper row: mature spikes of a naked grain T0 plant (T-A4, left) compared to a wild-type hulled plant of the corresponding cultivar Tselinniy 5 (right). The hull-less phenotype is expressed along the entire spike of the edited plant; however, it is most clearly visible in the regions where the husk has opened more widely, and is less apparent in the regions where the husk segments remain tightly appressed. Arrows indicate the regions where the easily detachable husk characteristic of the hull-less phenotype is most clearly observed. The lower panel shows seeds harvested from the corresponding spikes after threshing.

T_0_ plants displayed various allelic configurations, with most edited plants being biallelic. Eight plants carried biallelic mutations, one plant was heterozygous (T-C2), and one was homozygous (T-C8). These results are consistent with previously reported editing efficiencies of the Nud45 and Nud50 gRNAs (Gerasimova et al., 2018). In agreement with previously published data, frameshift mutations present in homozygous or biallelic states resulted in a naked grain (hull-less) phenotype in T_0_ (**Figure 6**). In contrast, plants carrying the wild-type *NUD* allele (e.g., A-302, G-310) or heterozygous plants (e.g., T-C2) retained the hulled phenotype; hulls could not be removed mechanically or manually.

### 3.4 Off-target analysis in T_0_ plants

According to Gerasimova et al. (2020), the Nud45 gRNA has three potential off-target loci in the barley genome located on chromosomes 2H, 6H, and 7H. The 2H locus corresponds to the *WIN1* gene, which is involved in epicuticular wax biosynthesis (Gerasimova et al., 2023). The remaining loci on chromosomes 6H and 7H are positioned within *AP2/ERF* genes of unknown function and share high sequence homology with *NUD*. Previous studies reported that the high activity of Nud45 was associated with unintended mutations at these loci. Although the Nud50 gRNA matches these genomic sequences, the absence of a flanking protospacer-adjacent motif (PAM) prevents Cas9 cleavage at these sites (Gerasimova et al., 2020).

In the present study, all T_0_ plants harboring the JD633 vector, as well as those lacking detectable vector integration but carrying *NUD* mutations, were screened for mutations at the three Nud45 off-target loci. Mutations were detected at all three loci in most on-target mutant plants, with the exception of plant T-C2, which carried a heterozygous *NUD* mutation and showed no off-target edits. Plants carrying homozygous (G-309) or biallelic mutations (T-C8, T-D8, A-301, A-322, G-324) in the *WIN1* gene exhibited a visible waxless phenotype (**Supplementary Figure 3**). Heterozygous mutations in *WIN1* were observed in plants G-303 and G-321, which showed no mutations in the target *NUD* gene or at the 6H and 7H off-target loci (**Supplementary Table 5**). Plants T-331 and A-315 harbored more than two alleles at the *WIN1* locus, whereas T-C8, T-D8, and T-331 exhibited multiple alleles at the 6H locus, consistent with a chimeric state (**Supplementary Table 5**).

Comparison of mutation profiles enabled identification of probable clonal regenerants. Plants G-303 and G-321, as well as T-C8 and T-D8, shared common callus origin and identical mutation patterns across all analyzed loci. Other plants displayed partial overlap in mutation profiles; for example, T-C8/T-D8 and T-331 shared identical mutations at the 7H and *WIN1* loci but differed in their *NUD* edits. While isolated single-nucleotide indels may arise independently, the observed multi-locus mutation patterns suggest a shared clonal origin for several T_0_ lines.

Overall, 9 of all 11 *NUD* mutants (90%) carried edits at two or more off-target loci. In addition, off-target mutations were detected in three plants that contained vector integration but lacked target *NUD* edits. In this experiment, off-target editing was observed more frequently than on-target editing.

### 3.5 Evaluation of chimerism in regenerated T_0_ barley plants

In embryogenic tissue cultures, somatic embryos and regenerated plants do not necessarily originate from a single progenitor cell. Continued Cas9 activity during callus development and plant differentiation can generate *de novo* mutations and tissue chimerism, potentially producing additional allelic variants in the T_1_ generation (Kapusi et al., 2017; Miroshnichenko et al., 2022). To assess the extent of chimerism, T_0_ mutant plants of cv. Tselinniy 5 generated with the JD633 vector were analyzed. This cultivar was selected because it produced the largest number of regenerants derived from both shared and independent callus events, enabling comparison of mutational stability within and between tillers originating from a common embryogenic source.

Three of the five analyzed plants (T-C8, T-D8, and T-331) originated from the same callus fragment, whereas the remaining two were derived from independent calli. Genomic DNA was extracted separately from flag leaves of six individual tillers per plant (three tillers for T-331) at the heading stage. Target and off-target loci were sequenced, and allelic compositions are summarized in **Supplementary Table 5**; **Supplementary Figure 3**.

All five T_0_ plants exhibited evidence of chimerism. Plant T-C2 showed chimerism with respect to plasmid integration: although vector sequences were not detected at the seedling stage, they were identified in three of six tillers at heading stage. No differences in mutation profiles at target or off-target loci were observed among these tillers. Other plants demonstrated more complex chimeric patterns. In plant T-A4, three of the six tillers were homozygous for a 1-bp deletion in the *WIN1* gene and exhibited a waxless phenotype, whereas the remaining tillers were heterozygous for the same deletion and retained a normal waxy phenotype (**Supplementary Figure 4**). No intra-plant variation was found in the *NUD* gene or the 6H and 7H off-target sites in T-A4. Plants T-C8 and T-D8 carried three distinct alleles at the 6H off-target locus across all analyzed tillers while remaining uniform at other loci. Plant T-331 exhibited the highest level of chimerism; each of the three analyzed tillers possessed a unique combination of target and off-target mutations (**Supplementary Table 5**).

### 3.6 Analysis of T_1_ progeny

To assess the inheritance of CRISPR/Cas9-induced mutations and determine whether new editing events occurred after regeneration, a subset of T_1_ progeny (4–20 per T_0_ plant) from six independent T_0_ plants was analyzed. Seeds were collected from the main spikes of self-pollinated T0 plants to minimize potential transmission of chimerism. To generate the T_1_ generation from T-C2, 20 seeds were collected from a spike confirmed to lack vector integration. Leaf samples from 4–20 T_1_ progeny per T_0_ plant were analyzed by qPCR and sequencing.

Sequencing confirmed that biallelic T_0_ plants produced exclusively edited progeny carrying mutant alleles, identified in T_0_ plants. Progeny derived from a heterozygous T_0_ plants included wild-type, heterozygous, and homozygous edited genotypes. The observed T_1_ genotypes were consistent with those identified in the T_0_ generation, reflecting the expected inheritance pattern of the edited alleles.

Biolistic transformation is known to frequently produce higher transgene copy numbers than *Agrobacterium*-mediated transformation, which can complicate elimination of vector sequences in subsequent generations (Altpeter et al., 2016). Estimated transgene copy number in T_0_ plants varied substantially (**Supplementary Table 6**), typically ranging from 1 to approximately 180 copies. Apparent higher estimates likely reflected tissue chimerism. Despite this variation, plasmid-free edited individuals were recovered in the T_1_ generation for all three cultivars (**Table 4**).

**Table 4:** Identification of T1 mutants lacking detectable vector sequences by PCR and analysis of the hull-less phenotype in *NUD*-edited barley lines.

T_0_ plant	No. of analyzed T_1_ plants	No. of PCR- negative T_1_ plants *	Naked/Hulled T_1_ phenotype
T-А4	19	19	19/0
T-С2	20	20	20/0
A-301	5	2	5/0
A-388	4	2	2/2
G-309	11	2	11/0
G-324	7	5	7/0

* PCR screening was performed using primers specific for the *hptII* gene and the gRNA cassette. Plants negative in both assays were classified as lacking detectable vector sequences by PCR. The hull-less phenotype was scored visually on mature grains of T1 plants.

For example, T_0_ plant G-309 was estimated to contain ~50 integrated vector copies, yet two of eleven analyzed T_1_ progeny derived from this plant lacked detectable vector sequences. Similarly, T_0_ plant G-324 with an estimated ~20 copies produced five plasmid-free individuals among seven analyzed T_1_ progeny. Plasmid-free edited T_1_ plants were also identified among progeny derived from T_0_ plants A-301 and A-388. In contrast, T_0_ plants T-A4, as well as its T_1_ progeny showed no vector integration, consistent with editing arising from transient CRISPR/Cas9 expression. Regarding T-C2, although vector sequences were detected in some tillers of the T_0_ plant (Section 3.5), the selected spike was plasmid-free, resulting in a T_1_ progeny lacking vector integration. No novel mutations were detected in T_1_ plants, including those retaining vector sequences. Moreover, chimerism was not observed in the analyzed T_1_ individuals. For instance, T_1_ progeny of plant A-315 segregated uniformly for the *WIN1* phenotype, indicating that the chimerism observed in the parental T_0_ plant was somatic and not inherited.

## 4 Discussion

### 4.1 *TaGRF4-GIF1* chimera enables barley transformation, regeneration, and genome editing in commercial cultivars

Despite extensive efforts to optimize barley transformation protocols and the advances in genome editing, genotype dependency remains a major limitation in cereal genetic engineering (Orman-Ligeza et al., 2020; Luo et al., 2025). Most targeted genome editing studies in barley continue to rely on the model cultivar Golden Promise, which exhibits exceptional *in vitro* regeneration capacity and high amenability to *Agrobacterium*-mediated transformation (Bekalu et al., 2023; Hanak et al., 2023). In contrast, efficient editing of non-model cultivars remains comparatively limited (Lim et al., 2018; Tiwari et al., 2022; Wang et al., 2023). This recalcitrance extends even to cultivars such as Morex, which possess high regenerative potential yet remain difficult to transform using *Agrobacterium*-mediated delivery (Hensel et al., 2008; Hisano and Sato, 2016; Orman-Ligeza et al., 2020).

Transformation and editing across diverse genetic backgrounds is essential because cultivars differ in regulatory architecture and allelic composition. Previous strategies to overcome genotype dependency have involved either introgression of target loci into Golden Promise or transfer of *Transformation Amenability* (*TRA*) loci from Golden Promise into other cultivars (Deng et al., 2015; Sato et al., 2016; Orman-Ligeza et al., 2020; Buchmann et al., 2025). Although effective, these approaches are time-intensive and may introduce linkage drag of undesirable genetic material. Moreover, emerging editing techniques – such as base editing or prime editing – require substantially higher transformation throughput than is currently achievable in most barley cultivars.

Recent strategies have therefore focused on morphogenic regulators capable of bypassing endogenous developmental barriers. These regulators promote cellular reprogramming by enabling somatic tissues to acquire embryonic competence (Lian et al., 2022; Niu et al., 2026). Several morphogenic regulator genes have been employed in cereal transformation, including *WUSCHEL* (*WUS*), *BABY BOOM* (*BBM*), *WUSCHEL*-related homeobox (*WOX*) genes, and the *GRF4-GIF1* chimera. *WUS*- and *BBM/WUS*-based systems substantially enhance regeneration and transformation efficiency in monocots: in wheat, *BBM/WUS* expression increased transformation efficiency five- to sixfold (Zhou et al., 2022), and in barley, a threefold increase was observed with the *PLTPpro: HvBBM* + *Axig1pro:HvWUS* construct (Suo et al., 2021). Similarly, *TaWOX5* enabled transformation of 31 common wheat cultivars, with transformation efficiencies reaching 90–100% in several varieties and 78.6 ± 9.8% in the barley cultivar Vlamingh, compared with 10.1 ± 4.3% for the control vector (Wang et al., 2022). However, constitutive or unregulated expression of *WUS*, *BBM/WUS*, and related transcription factors has frequently been associated with substantial pleiotropic effects, including malformed shoot morphology, sterility, and tumor-like overgrowths, necessitating excision of the morphogen after the induction of somatic embryos or meristem development (Lowe et al., 2016; Maren et al., 2022; Lee and Wang, 2023; Chaudhary et al., 2026). For *WOX5*, pleiotropic effects have also been documented. Wang et al. (2022) reported wide, short flag leaves and thick stems in *WOX5*-expressing plants, but the phenotype of mature plant was not described. Hayta et al. (2026) further showed that *WOX5* expression caused approximately 41% sterility, wider spiraled leaves, increased grain area, weight, and length, and reduced tiller and spike number (Hayta et al., 2026). In contrast, the *GRF4-GIF1* chimera enhances regeneration and transformation efficiency with comparatively fewer pleiotropic effects. The average regeneration efficiency with *GRF4-GIF1* (65.1 ± 5.0%) was 7.8-fold higher than that of the empty vector control (8.3 ± 1.9%), and the construct enabled transformation across bread wheat, rice, and triticale varieties without the severe developmental abnormalities associated with *WUS/WOX*-based systems (Debernardi et al., 2020). These considerations, together with the limited availability of barley-specific data at the time of experimental planning, guided our selection of *TaGRF4-GIF1* as a morphogenic regulator offering a more favorable balance between regeneration capacity and phenotypic normality. A recent study in barley demonstrated that the *HvWUS* + *HvBBM2* combination is also a promising morphogenic system, providing high transformation efficiency without compromising overall plant growth (He et al., 2025). The authors reported some effects on grain number per spike and thousand-grain weight; however, these alterations were relatively minor compared with the severe developmental abnormalities documented for *WUS/WOX*-based systems in other species. Notably, comparable effects on grain number have also been reported for *GRF4-GIF1* in cereals (Debernardi et al., 2020).

In the present study, expression of the wheat-derived *TaGRF4-GIF1* chimera enabled stable biolistic transformation in three commercial barley cultivars Tselinniy 5, Aley, and G-23035 that were otherwise recalcitrant under the tested conditions. Standard scutellum-based transformation protocols optimized for Golden Promise (Marthe et al., 2015) repeatedly failed in cultivars Tselinniy 5, Aley, and G-23035 in our laboratory despite multiple independent experiments. Under the conditions used in this study, no stable transformants were recovered with the control vector lacking the morphogenic regulator, whereas the JD633 (*TaGRF4-GIF1*) construct supported the recovery of stable transgenic regenerants. This observation is consistent with the limited responsiveness of these cultivars to conventional transformation protocols and provides the rationale for evaluating the *TaGRF4-GIF1* chimera in this context.

We acknowledge that the use of different selection systems in our work may have influenced the stable transformation frequencies observed. Different selectable markers (*bar* in the L2 vector and *hptII* in JD633) impose distinct selection pressures. Hygromycin is currently the preferred selective agent for stable transformant recovery in the standard *Agrobacterium*-mediated transformation protocol for the barley cultivar Golden Promise. However, before this protocol was established, phosphinothricin and its analogues (bialaphos and Basta) were widely used for biolistic barley transformation, enabling transformation of Golden Promise and several other cultivars, although at low efficiencies ranging from 0.1 to 1.6% (Lemaux et al., 1999). A later study reported higher transformation efficiency using phosphinothricin and biolistic delivery in cultivar Colon (2.2%) (Tobias et al., 2007).

Because the experimental JD633 and control L2 constructs differed in their selectable marker systems, we cannot attribute the improved transformation efficiency observed with JD633 solely to the *GRF4-GIF1* morphogenic regulator. The possibility that differences in selection pressure contributed to the observed differences cannot be excluded. Nevertheless, as noted above, stable transformants were recovered exclusively with the JD633 construct across all three tested cultivars, which suggests that the morphogenic regulator facilitated extending transformation competence, although the relative contributions of *GRF4-GIF1* and the selection system cannot be distinguished within the present experimental design. Future studies comparing constructs that differ only in the presence or absence of *GRF4-GIF1*, while using identical selectable markers, will be required to rigorously assess the contribution of the morphogenic regulator.

Transformation efficiencies approaching 5% in non-model barley cultivars represent a practical advance, even if lower than those typically reported for the model cultivar Golden Promise (10–25%). Such efficiencies are sufficient to support direct genome editing in elite germplasm, reducing the need for prolonged backcrossing. While introgression strategies remain useful, they are laborious and may retain unwanted linked loci. Our results are consistent with previous reports across monocot crops – including wheat, triticale, rice, maize, sorghum, and oat – where *TaGRF4-GIF1* improved transformation and editing efficiencies (Debernardi et al., 2020; Li et al., 2024; Vandeputte et al., 2024; Mehtab-Singh et al., 2025). For example, the *TaGRF4-GIF1* chimera increased regeneration efficiency in durum and common wheat by up to 7.8-fold, corresponding to a genome editing success rate of 93.7% (Debernardi et al., 2020). In sorghum, the *TaGRF4*-*OsGIF1* fusion increased transformation efficiency by 7.71-fold compared with the original protocol (Li et al., 2024).

The function of GRF transcription factors is negatively regulated at the post-transcriptional level by the microRNA miR396. Together with their GIF co-activators, GRFs form the conserved miR396–GRF/GIF regulatory module that coordinates cell proliferation and differentiation during plant development (Debernardi et al., 2014; Liebsch and Palatnik, 2020). In the original work of Debernardi et al. (2020), a miR396-resistant version of the GRF-GIF chimera was engineered only for *Vitis* transformation, whereas the wheat TaGRF4-GIF1 chimera was designed without modifying the miR396-binding site. Thus, the chimeric transcript delivered by JD633 remains, in principle, susceptible to miR396-mediated cleavage. Nevertheless, the wild-type TaGRF4-GIF1 chimera was functional in our experiments. We attribute this to the high constitutive expression driven by *pZmUbi* promoter, which ensures that sufficient intact mRNA is produced despite miR396-mediated cleavage of a fraction of the transcripts. Qiu et al. (2022) demonstrated that introducing synonymous mutations into the miR396 target site of TaGRF4 (mTaGRF4-GIF1) further improved regeneration and genome editing efficiency in wheat compared with the original TaGRF4-GIF1 chimera (Qiu et al., 2022). These findings suggest that the wild-type GRF4-GIF1 chimera remains functional despite partial miR396-mediated repression and that engineering miR396 resistance represents a promising strategy for further optimization. *GRF-GIF* modules have also been reported to enhance regeneration in dicotyledonous species, including several transformation-recalcitrant crops such as strawberry, watermelon, *Beta vulgaris*, *Helianthus annuus*, *Brassica napus*, *Citrullus lanatus*, Citrus spp., *Lactuca sativa* (Niu et al., 2026). In *Solanum lycopersicum*, the *SlGRF4c-GIF1a* chimera increased explant regeneration efficiency by 1.8-fold, raising overall transformation frequencies from 7% to 13% and shortening the regeneration cycle by approximately four weeks (Swinnen et al., 2025). Reports in cereals indicate that pleiotropic or developmental effects associated with *GRF* and *GIF* genes families are generally limited compared with other morphogenic regulators (Debernardi et al., 2020; Li et al., 2024). In contrast, such effects appear more frequently in dicots, suggesting evolutionary lineage-dependent differences in transgene regulation and developmental response (Debernardi et al., 2014; Lv et al., 2023; Sanchez et al., 2024).

In our study, the use of *GRF4-GIF1* did not produce evident negative effects on subsequent plant growth and development in the three barley cultivars examined. Although pleiotropic changes were observed at the callus stage, almost all regenerated plants reached maturity and produced viable T1 seeds, with only a few exceptions (four plants) described in **Supplementary Table 5** and the Discussion. These observations indicate that, within the limited set of genotypes tested here, morphogen-associated developmental alterations were transient and did not prevent the recovery of a normal phenotype or fertility. However, given that only three barley cultivars were evaluated, and in light of the genotype-specific pleiotropic effects of *GRF4-GIF1* reported by Hayta et al. (2026) across hexaploid and tetraploid wheat, we cannot exclude the possibility that other barley genotypes may respond differently. Further evaluation across diverse barley germplasm will be required to assess the generality of these observations.

Our observations in barley differ from the copy-number-dependent sterility effects reported in wheat by Hayta et al. (2026). Whereas sterility in wheat was supposed to be associated with high transgene copy numbers, no comparable relationship was detected in our material. We successfully recovered fertile plants across a wide range of integration events (1 to about 80 copies), and sterility was observed only sporadically (e.g., in individual plants with 10 and approximately 180 copies). These findings do not support a strong copy-number-dependent sterility effect in the barley genotypes tested, although the limited sample size precludes definitive conclusions, and instead suggest that the rare sterility events observed here are more consistent with stochastic position effects or somaclonal variation than with a direct gene-dosage response. However, this interpretation is based on a limited sample and should be verified in larger populations.

To date, only a limited number of studies have examined the use of *TaGRF4-GIF1* for barley transformation. Prusty et al. (2025) reported reduced regeneration efficiency of transformed immature embryos in the model cultivar Golden Promise when the chimera was introduced, despite higher initial transformation signals based on a reporter assay. The authors suggested that *GRF4-GIF1* overexpression may interfere with endogenous regeneration pathways in barley. One possible interpretation is that this response reflects genotype-specific effects, as Golden Promise already exhibits high intrinsic regeneration capacity. The relatively small experimental scale of that study (20 embryos in a single replicate) also indicates that additional validation under broader conditions would be informative. Similarly, Yang et al. (2022) reported recovery of a limited number of transgenic plants (only six T_0_ individuals) following *GRF4-GIF1*-mediated transformation in Golden Promise, whereas standard protocols without morphogens typically yield tens to hundreds of T_0_ regenerants. This result suggests that for genotypes with already high regeneration capacity, the strong constitutive expression of morphogenic factors under strong promoters, such as *ZmUbi* might be redundant or even inhibitory. In such cases, the excessive presence of morphogenic factors may disrupt the normal transition of cells through the regeneration stages, leading to a lower output compared to established protocols. Further studies using tissue-specific or inducible promoters may be needed to clarify this effect and optimize the use of morphogenetic regulators in different barley varieties.

While direct comparisons across studies are complicated by methodological differences, these observations raise the possibility that, morphogenic stimulation from the *GRF4-GIF1* chimera may interact differently with highly regeneration-competent genotypes. In such backgrounds, additional signaling input could approach or exceed optimal developmental thresholds, potentially affecting shoot recovery. In contrast, our results obtained in transformation-recalcitrant commercial cultivars indicate that *GRF4-GIF1* can facilitate stable regeneration under conditions where conventional protocols were ineffective. While highly regeneration-competent model genotypes may exhibit reduced regeneration efficiency when *GRF4-GIF1* is used, our results indicate that inclusion of the chimera can substantially improve stable transformation and genome editing in elite barley germplasm.

Although *TaGRF4-GIF1* genes are primarily known for their effect on regeneration capacity in recalcitrant genotypes, their indirect contribution to genome editing success lies in increasing the total number of independent transgenic events available for screening and selection of mutants. Because transformation efficiency was assessed based on the recovery of independent transgenic plants, the present study does not distinguish between effects on the initial transformation events and the subsequent regeneration of transformed cells.

The apparent discrepancy between the approximately 10–11% mutation frequency detected in transformed callus tissues during the preliminary assessment and the 64.3% genome editing efficiency observed among regenerated transgenic plants reflects the different biological stages and cell populations represented by these measurements. The mutation frequency in calli was estimated from bulk tissue containing a mixture of edited and non-edited cells, including cells with stable vector integration and Cas9 expression, cells with only transient Cas9 expression, and cells with no Cas9 expression. During subsequent selection and regeneration, cells that had stably integrated the vector DNA and expressed the Cas9/gRNA cassette were preferentially retained and gave rise to shoots, thereby enriching the regenerating population for transformed and, consequently, edited cells. The higher editing frequency observed at the plant level is therefore consistent with the enrichment of transformed cells during selection and regeneration, whereas the callus-level value reflects an earlier stage of the process in a heterogeneous cell population.

Analysis of the T_1_ generation confirmed stable inheritance of the edited *NUD* alleles across all six independent T_0_ lines, indicating that the editing outcomes generated by biolistic transformation were faithfully transmitted to the progeny. The naked grain phenotype observed in the T_0_ plants was likewise transmitted to the T_1_ generation, providing further evidence that the phenotype is causally linked to *NUD* editing rather than to T-DNA integration effects or tissue culture-induced somaclonal variation. In the T_1_ generation, all analyzed progeny of lines T-A4, T-C2, A-301, G-309, and G-324 displayed the hull-less phenotype, while line A-388 produced both hull-less and hulled progeny, consistent with the heterozygous state of the *NUD* allele. Notably, transgene-free edited T_1_ individuals were recovered from all three barley cultivars, including the progeny of T_0_ plants that had carried high estimated transgene copy numbers.

Importantly, no novel mutations or additional off-target editing events were detected in the analyzed T_1_ plants, and chimerism was not observed in the T_1_ generation, suggesting that the editing outcomes established in the T_0_ plants are stable during meiotic transmission. However, it should be acknowledged that not all T_1_ progeny were included in the analysis; instead, only a limited subset was examined (ranging from 4 to 20 T_1_ plants per line, depending on the T_0_ plant).

Consequently, while the available data support stable inheritance of the edited alleles and the associated hull-less phenotype, we cannot entirely exclude the possibility that novel mutations or additional off-target events may be present in unanalyzed T_1_ progeny. Larger-scale analyses of the T_1_ generation will be required in future studies to provide a more comprehensive assessment of editing stability across generations.

### 4.2 Optimization of DNA delivery and selection strategy

Transformation protocol parameters were optimized in a single cultivar before being extended to additional genotypes, an approach also employed in cereal transformation studies by He et al. (2025); Biswal et al. (2023); Debernardi et al. (2020), and others. Although this strategy does not guarantee that the optimized parameters are universally optimal, the successful transformation of all three cultivars using the same protocol supports its applicability across the tested germplasm.

The choice of explant represents a critical parameter for successful plant transformation. While many barley protocols rely on freshly dissected immature embryos (Kapusi et al., 2017; Bekalu et al., 2023), our results indicate that targeting one-week-old embryogenic callus improved recovery of stable transformants. The observed difference in transformation efficiency between immature embryos and IE-derived calli may be attributable to several factors. Immature embryos may be more susceptible to mechanical damage caused by gold particles, potentially exceeding their capacity for physiological recovery and reducing their regenerative competence. In contrast, proliferating callus tissue possesses a larger population of undifferentiated cells, which may better tolerate the mechanical stress associated with biolistic delivery. Further optimization of biolistic parameters, such as rupture disk pressure, target distance, and microcarrier density, may improve embryo-based transformation efficiency in future studies.

Callus tissue that has already initiated an active proliferative state appeared more receptive to the modified biolistic procedure. The advantage of embryogenic callus has been reported in other transformation-recalcitrant cereals, such as emmer wheat (*T. dicoccum*) or oat (*Avena sativa L.*), where bombardment of compact nodular calli supported stable transformation (Miroshnichenko et al., 2020; Mehtab-Singh et al., 2025).

Although *GRF4-GIF1* has been proposed to reduce the duration of the regeneration process in several species, the present protocol still relied on a short (6–8-day) immature embryo-derived callus stage because this explant type provided substantially higher regeneration efficiency after biolistic damage than freshly isolated immature embryos in the elite barley cultivars used in this study. We did not directly evaluate the frequency of somaclonal variation and therefore cannot determine the extent to which the brief callus phase contributed to genomic variation. Future genome-wide analyses will be required to compare the genetic stability of regenerants obtained from immature embryos and IE-derived calli.

Particle bombardment can simultaneously deliver DNA to multiple cells within a single explant. As a result, a single scutellum-derived callus may give rise to several independent cellular clones, each capable of producing a regenerated plant. Consequently, multiple independent transgenic lines can originate from a single explant, whereas multiple regenerants from the same callus may share a clonal origin. In the absence of Southern blot analysis, flanking PCR, or adaptor-ligation-mediated PCR, which provide more direct molecular characterization of transgene integration events and were not performed in this study, the use of mutation profiles at the *NUD* target and off-target loci represents a pragmatic approach to distinguish independent events, based on the principle that identical NHEJ outcomes at multiple loci are unlikely to arise independently. However, we cannot entirely exclude the possibility that some events classified as independent may in fact represent clonal transformations with different editing outcomes or, conversely, that some events classified as clonal may represent independent transformations that coincidentally produced identical editing patterns. More detailed molecular characterization of transgene integration events will be the focus of future studies.

An additional technical modification involved the use of the cationic lipid reagent TransIT-2020 for plasmid DNA coating. Conventional spermidine/CaCl_2_ precipitation remains widely used for DNA coating to gold microcarriers. However, lipid-based reagents, such as TransIT-2020 and Lipofectamine, have emerged as alternative delivery systems for DNA and RNP complexes in plant tissues (Svitashev et al., 2016; Qiu et al., 2025; Thorpe et al., 2025). Lipofectamine has also been used for protoplast transfection and for wheat embryo bombardment (Qiu et al., 2025). In this study, we evaluated both TransIT-2020 and Lipofectamine in comparison with the conventional spermidine-based coating protocol. In our comparison, all tested reagents supported transient GFP expression, but reproducibility differed. Spermidine-mediated coating produced variable fluorescence densities among biological replicates, whereas TransIT-2020 yielded more uniform signal distribution across treated calli. This consistency was associated with reduced variation in callus transformation efficiency (**Figure 3**). Although stable transformation frequencies did not differ statistically among reagents, the reproducibility and handling consistency of TransIT-2020 supported its adoption in the optimized protocol, in agreement with previous reports (Miller et al., 2021).

The vectors used in this work were originally constructed for *Agrobacterium*-mediated transformation; however, this did not limit their application in a biolistic system. Since DNA introduced by particle bombardment enters plant cells independently of the *Agrobacterium* Vir machinery, the T-DNA border sequences are not expected to contribute to DNA transfer. An advantage of particle bombardment is its compatibility with a broad range of plasmid constructs, allowing previously validated binary vectors to be tested without additional vector reconstruction. This flexibility simplifies the evaluation of existing genome-editing systems across different transformation approaches. Further improvements to the protocol may focus on delivery methods that favor transient expression of the CRISPR/Cas reagents, thereby reducing the probability of stable integration of vector-derived sequences.

Selection strategy may also influence recovery of regenerated plants. For example, the model cultivar Golden Promise has been reported to exhibit relatively high tolerance to high concentration of hygromycin (50 mg/l), with up to 23% of regenerants classified as non-transformed escapes (Holme et al., 2008; Waheed et al., 2016). In contrast, non-model cultivars may display greater sensitivity to hygromycin, as observed in other cereal systems. For instance, in indica rice, which is usually more recalcitrant than the model japonica varieties, necrosis of non-transgenic tissues can release inhibitory metabolites that hinder the recovery of transformed cells (Feng et al., 2021). Accordingly, Hiei and Komari (2008) recommend adjusting hygromycin concentrations for different rice cultivars (Hiei and Komari, 2008). In this study, we reduced the hygromycin concentration to 35 mg/L as a compromise between maintaining selection pressure and minimizing the toxic cross-effect of necrotic tissues on more sensitive non-model tissues. While this moderate pressure resulted in approximately 50% non-transgenic escapes, it effectively enabled the survival and recovery of stable transgenic events that might otherwise have been lost at higher concentrations. Additionally, the survival of non-transformed regenerants may be attributed to metabolic support provided by neighboring transgenic cells nursing effect previously described in barley transformation protocols, for example by (Bartlett et al., 2008).

Despite stringent selection, two regenerated plants carried on- and off-target mutations while lacking detectable vector integration. These individuals likely originated from cells in which CRISPR/Cas9 and *GRF4-GIF1* were transiently expressed, allowing genomic modifications to occurred before vector DNA was lost or degraded. Their survival under selection may reflect metabolic support from surrounding transformed cells within the callus mass. Following transfer to non-selective rooting medium, these transiently edited tissues regenerated into plants. Similar observations have been reported previously (Qiu et al., 2022; Kiseleva et al., 2025; Swinnen et al., 2025). Recovery of edited, vector-free individuals at the T_0_ is advantageous because it reduces the need for segregation in subsequent progeny.

It should be noted that the identification of plants lacking vector sequences was based on diagnostic PCR assays targeting multiple vector-specific regions. Although this approach is widely used for screening, it cannot completely exclude partial plasmid integration, small vector fragments integration, or chromosomal rearrangements that may occur following biolistic transformation. Definitive confirmation of plasmid-free status would require additional analyses, such as Southern blotting, long-read sequencing, or whole-genome sequencing.

Although the protocol enabled the recovery of independent transformants in four biological replicates and across three recalcitrant barley cultivars, the absolute numbers of regenerated plants and low-copy events remained limited. Therefore, the reported transformation frequencies should be regarded as preliminary estimates, and larger-scale experiments will be required to assess their precision and the reproducibility of low-copy event recovery.

### 4.3 Off-target effects and somatic chimerism

Implementation of the optimized transformation system required evaluation of editing precision and genomic stability. Previous studies indicate that Cas9-induced off-target mutation rates are generally low relative to genetic background variation arising from somaclonal processes. For example, in rice edited with 12 different gRNAs, off-target mutations were identified in only 2 of 47 plants (Tang et al., 2018). Similarly, in maize, off-target activity was primarily observed when gRNAs were intentionally selected for their high non-specific potential (Young et al., 2019). These findings support the view that well-designed gRNA typically produce limited off-target effects relative to natural genomic variability (Sturme et al., 2022).

Consistent with these reports, off-target editing in our material was confined to loci with complete sequence similarity to gRNA-Nud45+PAM, which had previously been identified as susceptible sites (Gerasimova et al., 2020). As whole-genome sequencing was not performed, the presence of off-target mutations in other genomic regions cannot be definitively excluded. Nevertheless, the observed mutation patterns and normal phenotype of the mutants align with previous findings suggesting that Cas9 activity outside of high-homology sites is relatively rare in cereals.

To evaluate genomic uniformity and potential chimerism, we performed detailed allelic analyses of five T_0_ plants from cultivar Tselinniy 5. Six independent tillers per plant at the heading stage were examined and compared with earlier seedling-stage genotyping. All analyzed plants were chimeric.

Prolonged Cas9 expression has been reported to generate distinct mutations in daughter cells within a single transformation event, resulting in somatic chimerism or mosaicism that can be transmitted to T_1_ progeny (Liu et al., 2020; Zeng et al., 2020; Miroshnichenko et al., 2022; Zang et al., 2022). For example, Zeng et al. (2020) reported emergence of additional mutation types at the heading stage in a substantial fraction of edited T_0_ barley plants. We propose that the elevated chimerism observed in this study may result from the combined effect of constitutive *Cas9* expression and the accelerated cell division and regeneration process associated with *GRF4-GIF1* activity. Rapid induction of somatic embryos and shoot primordia may permit incorporation of multiple cell lineages originated from a single transformation event, in which independent editing occurred before establishment of the shoot apical meristem (SAM). In contrast, conventional regeneration pathways are often dominated by a single progenitor cell lineage during meristem formation. Morphogen-stimulated regeneration associated with *GRF4-GIF1* activity may therefore favor simultaneous contribution of multiple edited cell lineages, each potentially harboring unique mutations.

Consistent with this interpretation, seeds collected from different tillers of the same T_0_ plant displayed increased allelic diversity in T_1_ progeny. Although somatic chimerism is frequently regarded as a complication due to early genotyping, it may also expand the range of edited alleles recovered from a single transgenic event. This increased variation may improve the likelihood of recovering desirable homozygous mutants in the T_1_ generation without requiring large numbers of independent transformants.

Our results emphasize the importance of a comprehensive genotyping approach in the T_0_ generation, particularly when using morphogenic regulators. High levels of somatic chimerism, which was potentially enhanced by *GRF4-GIF1*-stimulated cell proliferation, suggest that early-stage single-leaf analysis may not fully capture the allelic diversity within the plant. Therefore, screening multiple tillers at later developmental stages, such as heading, provides a more accurate representation of the edited lineages and their potential for germline transmission.

Although persistent Cas9 activity at later stages or in the T_1_ progeny has been reported in other systems (Miroshnichenko et al., 2022), the mutation spectra in our analyzed T_1_ plants remained consistent with those identified in their respective T_0_ parents. This conclusion is additionally supported by the analysis of off-target loci in the T_1_ progeny. The mutation profiles at the analyzed off-target sites remained to those of the T_0_ parents, with no novel off-target editing events. This suggests that the chimerism observed here primarily originated during early transformation events and was stably inherited. Such detailed T_0_ characterization supports more reliable predictions of genetic outcomes and facilitates the selection of desirable mutant alleles in the T_1_ generation.

### 4.4 Generation of plasmid-free edited lines

An important validation of this integrated approach is the ability to recover stable, plasmid-free mutant plants within an accelerated timeframe. One objective of this study was to enable efficient genome editing in commercial barley cultivars, which was achieved using the optimized protocol. Although biolistic transformation is often associated with high transgene copy numbers, we observed that transgene-free (null-segregant) plants could be recovered in the T_1_ generation even when the T_0_ plants carried high vector copy numbers (up to approximately 80 vector copies). Importantly, qPCR-derived copy number estimates quantify the abundance of vector-derived sequences rather than the number of independently segregating genetic loci. Therefore, relatively high estimated copy numbers do not necessarily preclude the recovery of transgene-free progeny following meiotic segregation.

Because only a limited number of T_1_ plants were analyzed for each family and copy number was estimated by qPCR, the precise chromosomal organization of the integrated vector sequences could not be determined. Nevertheless, the recovery of plasmid-free progeny from multiple independent events indicates that high qPCR-estimated copy numbers do not necessarily correspond to multiple independently segregating loci. Several non-mutually exclusive explanations may account for this observation. Complex integration structures, including tandem arrays and closely linked insertions, have previously been reported following biolistic transformation (Svitashev et al., 2002; Ozyigit and Kurtoglu, 2020; Smirnov and Battulin, 2021). Another possible explanation is that plants regenerated following biolistic transformation may be somatic chimeras with respect to integration events, and their germline may carry fewer integration loci than their somatic tissues. In this study, the relatively high proportion of transgene-free T_1_ individuals recovered from the A-301, A-388, G-309, and G-324 lines (**Table 4**) is consistent with integration at one or a small number of linked loci rather than with multiple unlinked integration events, although the limited family sizes do not allow formal genetic testing of this model.

Recovery of homozygous edited plants lacking vector sequences from relatively small T_1_ populations supports the practical feasibility of this strategy for rapid trait development. Accordingly, our strategy may enable development of edited commercial varieties (for example, a hull-less transgene-free line Tselinniy 5) within an accelerated timeframe of approximately 1.5–2 years. These findings suggest that incorporation of the *GRF4-GIF1* chimera has the potential to reduce genotype dependency in barley transformation and genome editing, providing a practical framework for precision breeding.

## 5 Conclusion

In this study, we evaluated the *GRF4-GIF1* morphogenic chimera in combination with an optimized biolistic workflow to address genotype dependency in barley genome editing. Our results demonstrate that this approach supported the transformation of three commercial cultivars that are difficult to transform. These findings indicate that *GRF4-GIF1* is an effective tool for enhancing cellular reprogramming across diverse barley germplasm, facilitating the recovery of transgenic plants from genotypes that previously yielded no transgenic events under our biolistic conditions. Technical refinements – including the use of one-week-old embryogenic callus and TransIT-2020 for DNA delivery – contributed to improved reproducibility of transformation.

Although morphogen-driven regeneration was associated with transient pleiotropic effects at the callus stage and somatic chimerism in T_0_ plants, these features expanded allelic diversity derived from individual transformation events. Recovery of homozygous, plasmid-free mutant plants in the T_1_ generation indicates that high-copy biolistic insertions do not preclude the recovery of transgene-free progeny, consistent with integration of the transgene cassettes at one or a small number of linked loci. Together, this accelerated workflow provides a foundation for the development of edited barley varieties within a shorter breeding timeframe.

Overall, this study establishes a practical genome editing framework that reduces genotype barriers and facilitates precision breeding in barley. The approach provides a scalable foundation for applying advanced editing technologies across commercially relevant genetic backgrounds.

## Data Availability

The original contributions presented in the study are included in the article/**Supplementary Material**, further inquiries can be directed to the corresponding author.
