# Supplementary material for: Impact of wheat GRF4-GIF1 morphogenic regulators on transformation and genome editing efficiency in elite barley cultivars
Source: Front Plant Sci. 2026 Aug 31;17:1837794. doi: 10.3389/fpls.2026.1837794 (PMC13572420; doi:10.3389/fpls.2026.1837794)
Supplement: Supplementary file 1 [file DataSheet1.pdf]

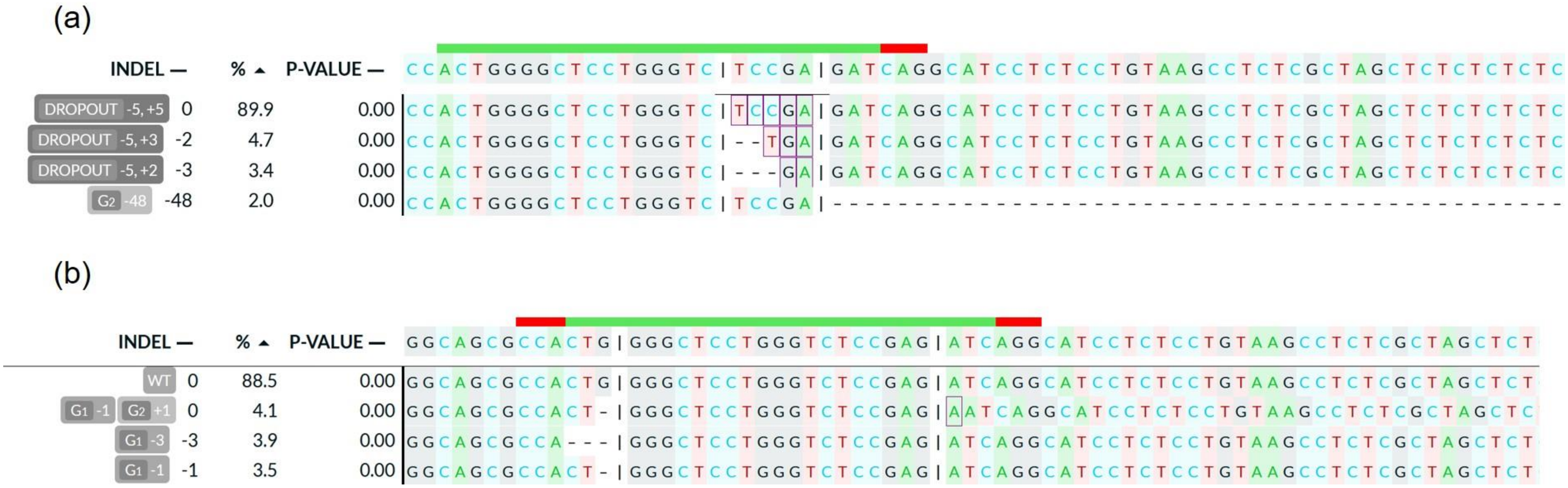

Figure S1. Sequences of the *NUD* gene in calli obtained after biolistic transformation of calli (a) and immature embryos (b). Forward and reverse Sanger sequencing reads for each sample were analysed using DECODR v3.0. The green line above the sequences indicates the gRNA target sites, and the red line indicates the PAM sites.

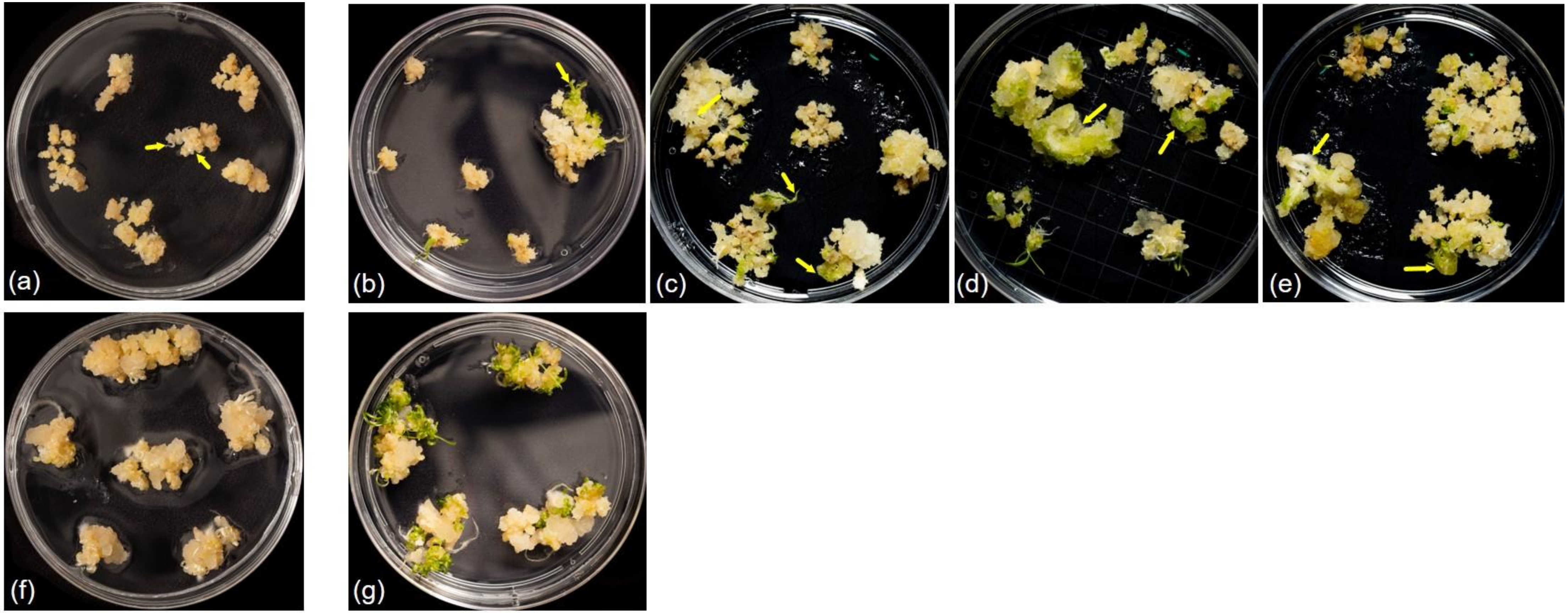

Figure S2. Morphological pleiotropic effects in calli transformed with the *GRF4-GIF1*-containing vector JD633. (a–e) Calli transformed with JD633 displaying developmental anomalies (indicated by arrows), including elongated filament-like protrusions and large, non-granular green tissue masses lacking typical embryogenic structure, (f–g) Control calli showing standard morphology. Images (a) and (f) show calli after 4 weeks on induction medium; images (b–e) and (g) show calli after two weeks on regeneration medium.

```
gRNA-45                    CCACTG GGGCTCCTGGGTCTCC
WIN1 wt   TCAGGCAGCGCCACTG GGGCTCCTGGGTCTCCGAG wt
T-A4_a1   TCAGGCAGCGCCACTG GGGCTCCTGGGTCTCCGAG wt
T-A4_a2   TCAGGCAGCGCCACT- GGGCTCCTGGGTCTCCGAG -1 bp
T-C2_a1   TCAGGCAGCGCCACTG GGGCTCCTGGGTCTCCGAG wt
T-C8_a1   TCAGGCAGCGCCACTGTGGGCTCCTGGGTCTCCGAG +1 bp
T-C8_a2   TCAGGCAGCGCCACT- GGGCTCCTGGGTCTCCGAG -1 bp
T-D8_a1   TCAGGCAGCGCCACTGTGGGCTCCTGGGTCTCCGAG +1 bp
T-D8_a2   TCAGGCAGCGCCACT- GGGCTCCTGGGTCTCCGAG -1 bp
T-331_a1  TCAGGCAGCGCCACTGTGGGCTCCTGGGTCTCCGAG +1 bp
T-331_a2  TCAGGCAGCGCCACT- GGGCTCCTGGGTCTCCGAG -1 bp
T-331_a3  TCA------------- -GGCTCCTGGGTCTCCGAG -14 bp
A-301_a1  TCAGGCAGCGCCACTGAGGGCTCCTGGGTCTCCGAG +1 bp
A-301_a2  TCAGGCAGCGCCACTGGGGGCTCCTGGGTCTCCGAG +1 bp
A-315_a1  TCAGGCAGCGCCACTG GGGCTCCTGGGTCTCCGAG wt
A-315_a2  TCAGGCAGCGCCACTGGGGGCTCCTGGGTCTCCGAG +1 bp
A-315_a3  TCAGGCAGCGCCACT-ins30-CTCCTGGGTCTCCGAG -4 bp>+30 bp
A-388_a1  TCAGGCAGCGCCACTG GGGCTCCTGGGTCTCCGAG wt
A-388_a2  TCAGGCAGCGCCACT-ins28-CTCCTGGGTCTCCGAG -4 bp>+28 bp
A-322_a1  TCAGGCAGCGCCACT- GGGCTCCTGGGTCTCCGAG -1 bp
A-322_a2  TCAGGCAGCGCCACT- -GGCTCCTGGGTCTCCGAG -2 bp
G-303_a1  TCAGGCAGCGCCACTG GGGCTCCTGGGTCTCCGAG wt
G-303_a2  TCAGGCAGCGCCACTGTGGGCTCCTGGGTCTCCGAG +1 bp
G-309_a1  TCAGGCAGCGCCACT- GGGCTCCTGGGTCTCCGAG -1 bp
G-321_a1  TCAGGCAGCGCCACTG GGGCTCCTGGGTCTCCGAG wt
G-321_a2  TCAGGCAGCGCCACTGTGGGCTCCTGGGTCTCCGAG +1 bp
G-324_a1  TCAGGCAGCGCCACT- -GGCTCCTGGGTCTCCGAG -2 bp
G-324_a2  ---------------- ----------------GAG -35 bp
```

Figure S3. Sequence analysis of the *WIN1* off-target locus in T_0 plants regenerated after transformation with JD633 carrying gRNAs Nud45 and Nud50. The *WIN1* locus was analyzed as one of the three predicted off-target sites of the Nud45 gRNA. Sequences in green boxes indicate the gRNA target sites, while sequences in grey boxes indicate the PAM motifs. Vertical dashed lines mark the predicted Cas9 cleavage sites. If a plant carried two or more distinct alleles, they were designated as a1 and a2 following the plant name. Mutations are shown in red.

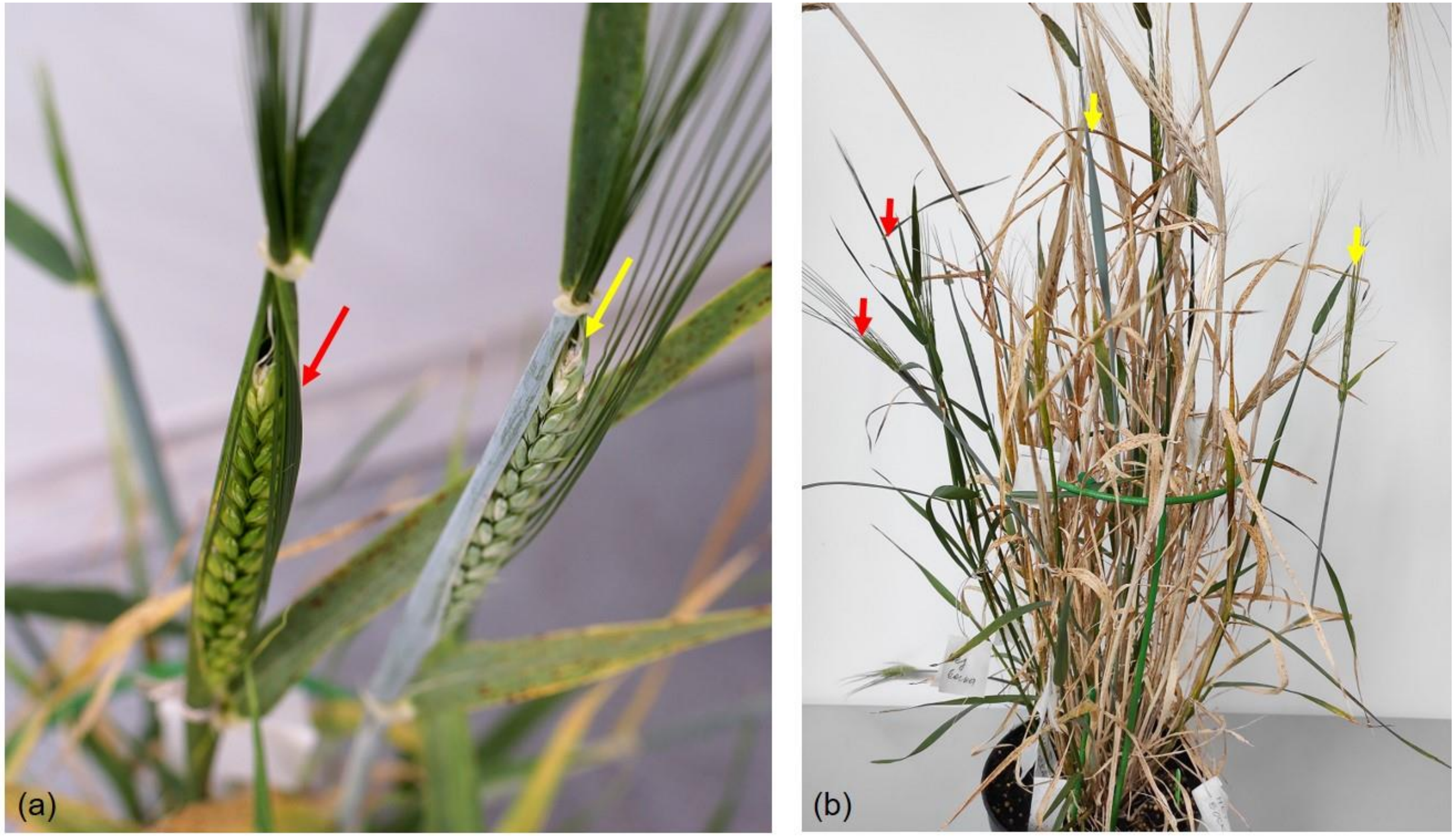

Figure S4. Phenotypic mosaicism in the edited T0 line. (a) Waxless spike phenotype (indicated by red arrows) resulting from a homozygous mutant *win1* allele in plant T-A4, compared to a wild-type spike (indicated by yellows arrow) from a different tiller of the same plant. (b) General view of the chimeric plant T-A4, where a single individual displays two distinct tiller phenotypes (indicated by red and yellow arrows).
